# Sandwich Miura‐Ori Enabled Large Area, Super Resolution Tactile Skin for Human–Machine Interactions

**DOI:** 10.1002/advs.202414580

**Published:** 2025-03-19

**Authors:** Qian Xu, Zhiwei Yang, Zhengjun Wang, Ruoqin Wang, Boyang Zhang, YikKin Cheung, Rui Jiao, Fan Shi, Wei Hong, Hongyu Yu

**Affiliations:** ^1^ Department of Mechanical and Aerospace Engineering The Hong Kong University of Science and Technology Kowloon Hong Kong SAR 999077 China; ^2^ Department of Mechanics and Aerospace Engineering Southern University of Science and Technology Shenzhen Guangdong Province 518055 China

**Keywords:** human–machine interactions, large area sensing, super resolution, tactile sensor

## Abstract

With substantial advances in materials science and electronics, flexible tactile sensors have emerged as a promising sector with extensive applications, notably in human‐machine interactions. However, achieving large‐area sensing with few sensing units at a low cost remains a challenge; the use of sensor arrays will complicate wiring and increase costs. To solve these issues, a sandwich Miura‐ori (SMo)‐enabled super‐resolution tactile skin capable of resolving normal and shear forces is proposed, and a theoretical model that incorporates the impact of actual manufacturing process is also developed, enabling the model to be employed for different tactile skins following calibration. Using machine learning techniques, the proposed tactile skin can accurately localize touch inputs (average localization error of 1.89 mm) and estimate the external force (average estimation error of 8%). Furthermore, a curved SMo skin is designed and fabricated using the tessellation algorithm, then installed on a robotic arm to control the motion, demonstrating its potential in human‐machine interactions. This research introduces a straightforward and cost‐effective approach to the design and manufacturing of super‐resolution tactile skins, and it also offers a valuable solution for future large‐area tactile sensor technologies.

## Graphical abstract

Sandwich Miura‐ori based tactile skin achieves large area sensing and super resolution, due to the interactions between different cells. It can also decouple external loads because of the special deformation mechanism. The curved tactile skin can be installed on robotic arms and achieve straightforward control and interactions, showing its great potential in human–machine interaction field.

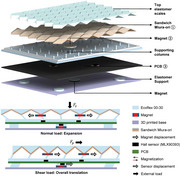

## 1 Introduction

Flexible tactile sensors is an emerging field with broad applications. Their deformability in various modes, including bending, stretching, and twisting, greatly enhances the environment–machine interfaces, making them vital in robotics and human–machine interactions. Numerous tactile sensors have been developed with various sensing technologies, such as magnetic field variation,^[^ ^1^, ^2^, ^3^, ^4^, ^5^ ^]^ magnetization‐based force decoupling,^[^ ^6^, ^7^ ^]^ electromagnetic induction,^[^ ^8^, ^9^ ^]^ resistance change and vibration,^[^ ^10^, ^11^ ^]^ and capacitance.^[^ ^12^ ^]^ Different functional materials, structures, and fabrication methods have also been used, including indium zinc oxide,^[^ ^13^ ^]^ transparent ionic materials,^[^ ^14^ ^]^ carbon quantum dots,^[^ ^15^ ^]^ Cu/Au nanolayers,^[^ ^16^ ^]^ carbon nanotubes (CNT) with PDMS,^[^ ^17^ ^]^ C‐type,^[^ ^18^ ^]^ multilayer,^[^ ^19^, ^20^ ^]^ cone‐shaped,^[^ ^21^ ^]^ and sponge‐like structures,^[^ ^22^ ^]^ as well as direct ink writing techniques^[^ ^23^ ^]^ for the manufacture of tactile sensors.

**Figure 1:**
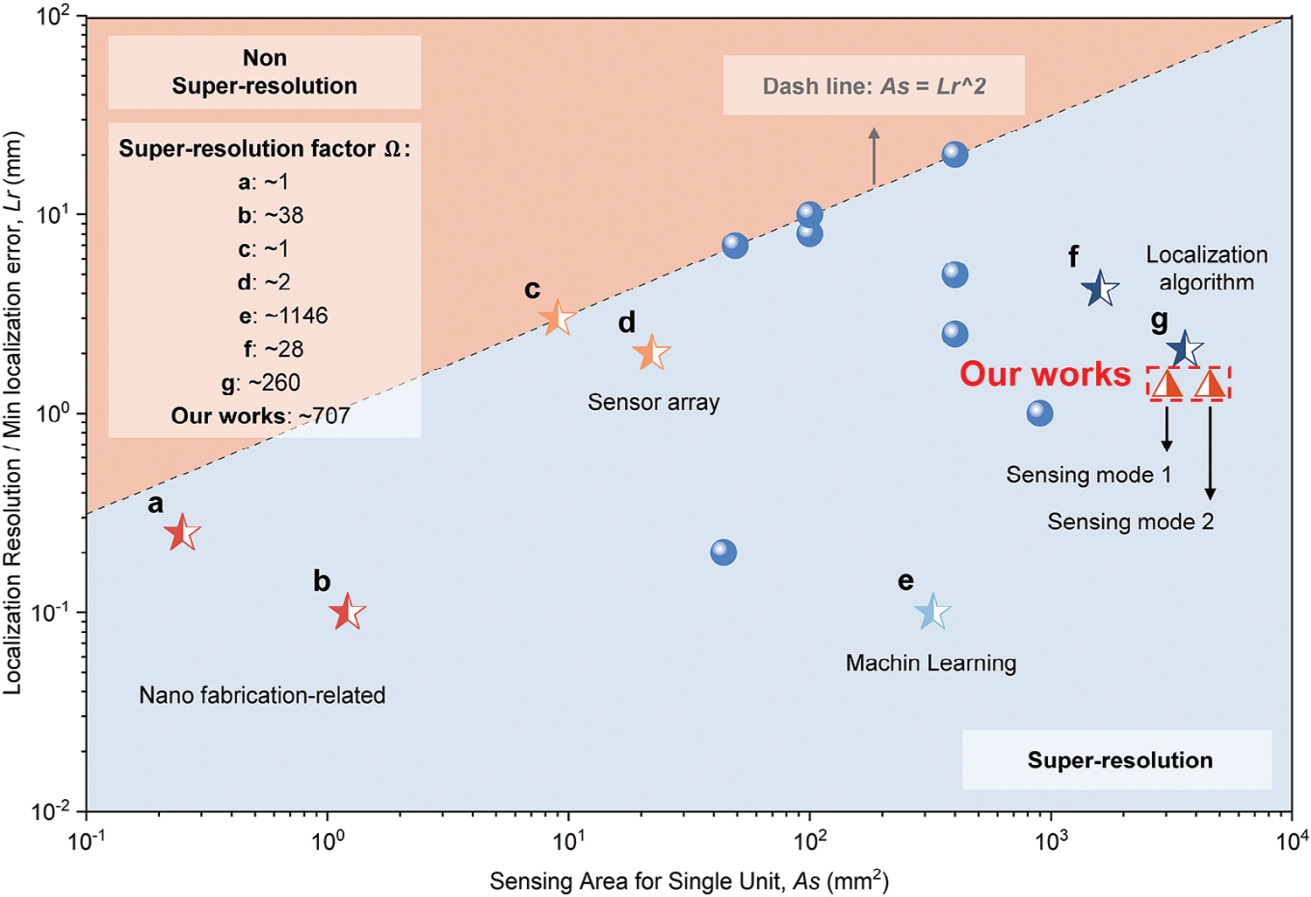
Typical examples of existing tactile sensors. a) Indium zinc oxide semiconductor nano‐membrane device.^[^ ^13^ ^]^ b) Origami structure inspired magnetic field sensor.^[^ ^1^ ^]^ c) Modular tactile sensor patch.^[^ ^26^ ^]^ d) Hybrid soft tactile sensor.^[^ ^27^ ^]^ e) Magnetic super resolution tactile skin.^[^ ^6^ ^]^ f) Multi‐layer‐structured biomimetic robotic skin.^[^ ^10^ ^]^ g) Wireless flexible magnetic tactile sensor.^[^ ^28^ ^]^ The localization resolution is the minimum distance that the sensor can recognize, sensing area for single unit (*As*) equals total sensing area divided by the number of sensing units, and super‐resolution is defined as *As* > *Lr* ^2^. Another definition about super‐resolution is the super‐resolution factor.^[^ ^29^ ^]^ Both definitions can be seen as the minimum distance that can be resolved by a single sensing unit. For our proposed SMo tactile skin, sensing mode 1 is for simultaneous measurement of normal force, shear force and localization, and sensing mode 2 is the pure normal force sensing and localization mode.

**Figure** **1** summarizes the performance of some existing tactile sensors, focusing on their relationship between localization resolution *Lr* (or minimum localization error) and the sensing area of a single unit *As*, showing the trend that the sensing area increases with increasing minimum localization error. Following the trend, sensors with accurate localization performance and small sensing areas^[^ ^1^, ^13^ ^]^ often require nanofabrication techniques. To increase the sensing area, sensor arrays are commonly used,^[^ ^10^, ^24^, ^25^ ^]^ leading to a more complex fabrication process, increased costs, and intricate wiring connections. In contrast, those with larger sensing areas^[^ ^10^, ^26^ ^]^ generally have a poorer localization performance. Sensors that deviate from the trend, such as the lower right corner, show that localization algorithms^[^ ^28^ ^]^ and machine learning^[^ ^6^ ^]^ can significantly improve localization performance. The super‐resolution factors Ω^[^ ^29^ ^]^ for these examples are also shown in the figure. For 2D case, Ω is proportional to the ratio of *As*/*Lr* ^2^. Different values of Ω illustrate again the importance of localization algorithms and machine learning techniques. From these examples, super‐resolution generally indicates that the deformation of structure or correlation of signals between different sensing units can be used to improve localization accuracy. However, some existing super‐resolution tactile sensors can only locate an external normal force without its magnitude information, or resolved normal and shear components. This severely limits the application scenarios and the ability of the sensors to cope with complex situations.

To address these challenges, we proposed a sandwich Miura‐ori (SMo)‐enabled, large‐area, super‐resolution tactile skin capable of locating and resolving external loads with a limited number of sensors. The Miura‐ori, a type of paper‐folding tessellation, has the property that the deformation of one cell can be propagated globally. Extensive research has been conducted on general Origami,^[^ ^30^, ^31^, ^32^ ^]^ Miura‐ori,^[^ ^33^, ^34^ ^]^ and other related structures,^[^ ^35^, ^36^, ^37^, ^38^ ^]^ as well as metamaterials^[^ ^39^, ^40^, ^41^ ^]^ and devices.^[^ ^42^, ^43^, ^44^ ^]^

In this paper, the SMo structure encodes the loads into the mutual and global displacements of vertices, allowing a large sensing area with fewer sensors, while the displacements are measured from the magnetic field readings. The special magnetization and the layered design facilitate effective resolution of external forces. We have also developed a theoretical model for the SMo tactile skin for rapid localization and straightforward external force estimation. The developed theoretical model takes into account the influence of the actual manufacturing process on the characteristics of the system, so that after calibration the model can be used for all tactile skins. By further implementing machine learning algorithms, the tactile skin achieved a more accurate localization and force estimation result. The performance and super‐resolution factor of the proposed SMo tactile skin are also shown in **Figure** 1. The SMo tactile skin has a larger *As* than most tactile sensors and a relatively high localization accuracy, corresponding to a large Ω. Finally, a curved skin was designed using a tessellation algorithm and integrated into a robotic arm. The tactile sensing signals were then used for the motion control of the robotic arm, allowing it to function as a robotic skin for interactions and collision protection.

## 2 Results

### 2.1 SMo Tactile Skin Structure and Design

**Figure 2:**
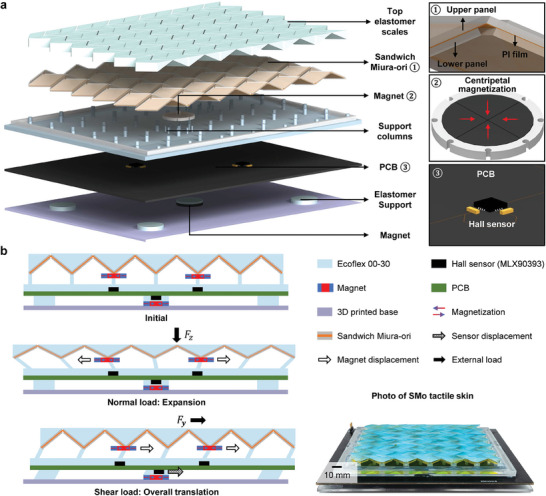
Structure and sensing mechanism of SMo‐enabled tactile skin. a) Structure of SMo tactile skin. Crease of SMo structure are optimized, and magnetic field signals are used as the readout of the structure deformation. b) Sensing mechanism of SMo tactile skin. Under normal loads, the SMo structure unfolds and the whole structure expands. Under shear loads, the whole structure translates globally due to the effect of top elastomer scales. In the sensing process, shear forces are measured at first by the lower Hall sensor, then the signals of two upper Hall sensors are corrected by the shear force to acquire the normal force induced variations, which is used for localization and normal force estimation.

The proposed SMo tactile skin consists of five layers: top elastomer scales, SMo structure, supporting columns, printed circuit board (PCB), and a bottom shear force sensing layer, as illustrated in **Figure** **2**a. The top elastomer scales were affixed to the SMo panels to ensure a flat contact surface, prevent folding of the SMo structure under shear loads, facilitate force resolution, and have a negligible influence on unfolding under normal load. The SMo structure comprises upper panels, a mid‐film, and lower panels. The supporting columns were bonded to the bottom vertices of the SMo structure. The PCB design featured three Hall sensors: two on the upper surface to locate external loads and estimate the magnitude of normal force, and one on the lower surface to measure the shear force.

The sensing mechanism is depicted in Figure 2b. Shear loads translate the entire structure on the feet globally, which the translation vector, and shear force can be measured by the lower Hall sensor. Under normal loads, the SMo structure unfolds. By analyzing the signals from these Hall sensors, the normal force can be located and estimated.

**Figure 3:**
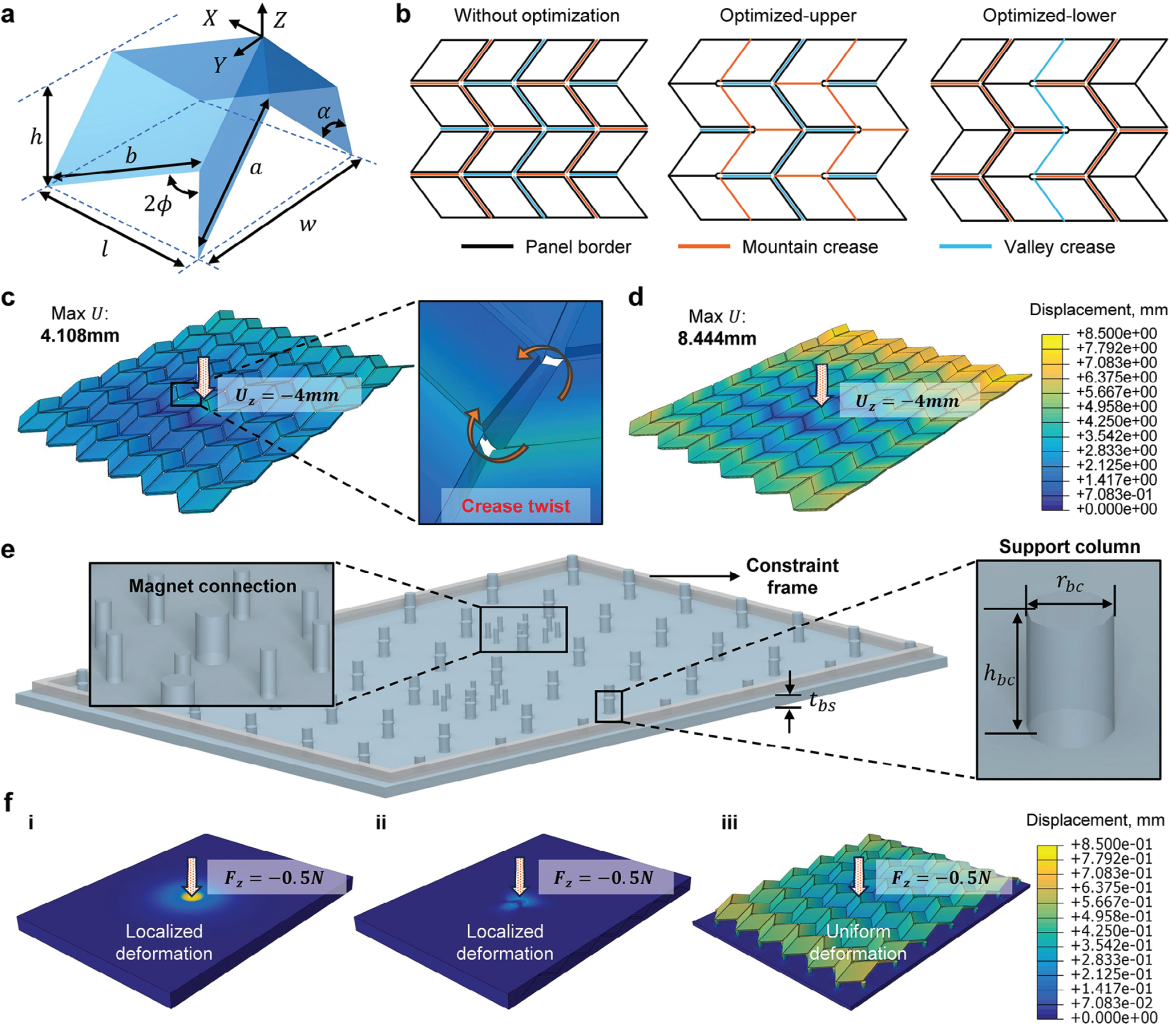
Geometric and mechanical design of SMo tactile skin. a) Geometric parameters of single cell. b) 2D pattern of Miura‐ori structure without crease optimization, optimized upper pattern and optimized lower pattern, respectively. c) Finite element analyses (FEA) of the SMo structure without crease optimization. A displacement boundary condition of *U*_*z*_ = −4 mm was given on top vertex of center cell. When without crease optimization, an obvious twist can be observed in the crease areas, which will hinder the propagation of deformation. d) FEA of the SMo structure with crease optimization. Same displacement boundary condition as (c) is applied. When crease is optimized, the folding and unfolding behaviors are quite close to the ideal rigid‐foldable case. e) Supporting columns design. f) FEA results for different package designs. (i) Elastomer cube, (ii) SMo structure fully encapsulated in elastomer, and (iii) Supporting columns design, respectively.

The geometric and structure design begins with an ideal rigid‐foldable Miura‐ori cell, as illustrated in **Figure** **3**a. The length, width and height of the cell are *l*, *w* and *h*, respectively. Panel lengths are *a* and *b*, and the panel interior angle is α, while the folding state angle is ϕ. For tactile sensing, our objective is to ensure uniform deformation in the two in‐plane directions (both *X* and *Y*) when load is applied. Therefore, the parameters were selected so that the out‐of‐plane Poisson's ratios are identical, ν_13_ = ν_23_:

(1) $$\begin{array}{ccc} -\frac{dl}{dh}\frac{h}{l} & = & -\frac{dw}{dh}\frac{h}{w}\;\Rightarrow \;\cot^{2}\phi \frac{\sin^{2}\alpha -\sin^{2}\phi }{\cos^{2}\alpha }\;\\ & = & \;\frac{\sin^{2}\alpha -\sin^{2}\phi }{\cos^{2}\alpha } \end{array}$$

which yields ϕ = π/4. Considering practical manufacturing processes, the lengths of the panels were set equal, *a* = *b* = 10 mm. The value of α influences *h*, ν_23_, and ν_13_, thus affecting the sensitivity. A larger α results in larger ν_23_ and ν_13_, as well as an increase in *h*. After balancing the Poisson's ratios and total thickness, α was selected as 50°.

To better approximate the folding and unfolding behaviors of ideal Miura‐ori, SMo structure with crease optimization was proposed. The SMo structure consists of three layers: upper panels, a middle film layer, lower panels. Crease optimization involves narrowing the mountain and valley creases in the upper and lower panels, respectively, as illustrated in Figure 3b. This optimized design significantly improves deformation propagation by minimizing crease areas, as shown by finite element analyses (FEA) Figure 3c,d. Without crease optimization, deformation transfer was hindered and energy was dissipated through twisting of the creases. In contrast, with crease optimization, more uniform deformation was observed in all cells.

Achieving tactile sensing solely with a Miura‐ori structure presents challenges. First, direct adherence to external surfaces is difficult. Second, a Miura‐ori structure would struggle to return to the original state by itself when released from stretching. Therefore, an array of elastomeric columns was introduced to support the SMo structure and bring it to the original state. The structure of the supporting columns is illustrated in Figure 3e. Each column has a height *h*_*bc*_ and radius *r*_*bc*_, and the columns are fabricated on a flat elastomeric substrate of thickness *t*_*bc*_. The selection of geometric parameters will be discussed later. These columns align with the bottom vertices of the Miura‐ori.

Figure 3f compares the FEA results of three different designs: a homogeneous layer of elastomer, a layer of elastomer embedded with a Miura‐ori structure, and a Miura‐ori structure supported by columns, when a load of 0.5 N is applied to the center of the top surface. The maximum displacement of the homogeneous elastomer layer was large, but the affected region was quite limited. When the Miura‐ori structure was fully encapsulated in elastomer, the elastic mismatch between the Miura‐ori structure and the elastomer significantly restricted the deformation, resulting in a small in‐plane displacement. In contrast, with the supporting columns, the maximum in‐plane displacement reached the order of 10^−1^ mm, 100 times higher than the fully encapsulated design. Additionally, the deformation was distributed over a much larger region compared to the other two designs.

### 2.2 Magnets Distribution and Localization

Magnetic flux density reading was used to measure the displacement because of ease of use, no direct contact, excellent stability, and high sensitivity. However, direct derivation of displacements and resolving external forces from variations in magnetic flux density often requires special magnetization such as Halbach arrays,^[^ ^6^ ^]^ but only two directions of magnetic field information were used. To fully exploit the magnetic field information, centripetal magnetization^[^ ^7^ ^]^ was adopted, as detailed in Section S2 (Supporting Information).

**Figure 4:**
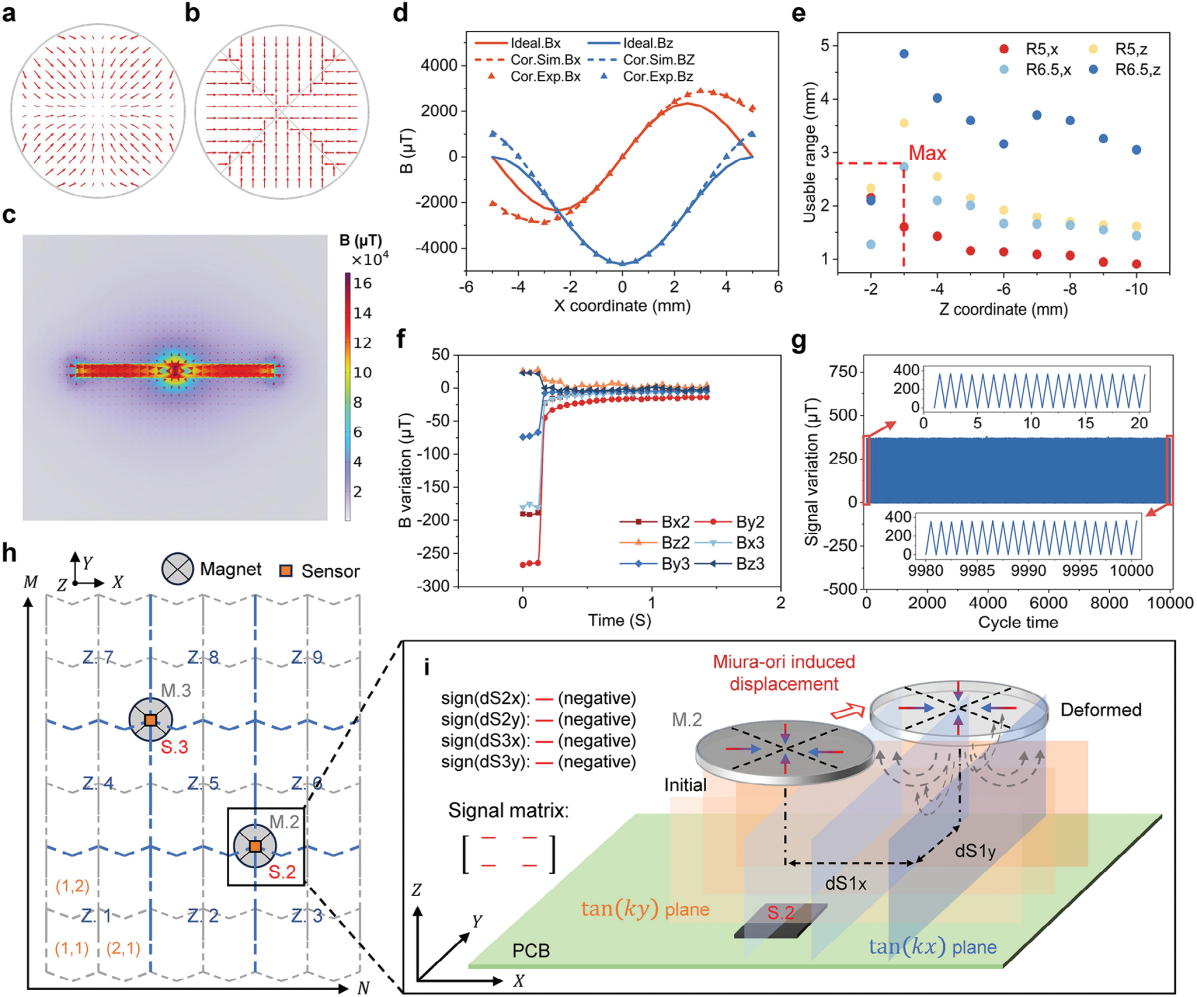
Magnetization and magnets distribution. a) Schematic of ideal centripetal magnetization, and b) Discrete magnetization. c) Magnetic flux density distribution of discrete magnetization on y‐z plane. d) Magnetic flux density comparison before and after correction. Ideal, Cor.sim. and Cor.Exp. indicate the ideal case, corrected simulation results and corrected experiment results, respectively. e) Usable measurement range after correction for different *z* coordinates. R5,x is the usable range in X direction for the magnet with radius of 5 mm, the rest are similar. f) Recovery time of upper two Hall sensors when a load of 1 N on cell (1,1) was removed. g) Repeatability test of SMo tactile skin. Here *B*_*x*2_ is shown, and similar results can be obtained for other signals. h) Cell numbering (1,1), (2,2)…, hall sensors (S.) and magnets (M.) distribution, and zone numbering (Z.). The lower magnet and Hall sensor, centered on the tactile skin, is omitted for clarity. i) Schematic of signal matrix when load on Zone 1.

The ideal centripetal magnetization (**Figure** **4**a) involves the superposition of two sinusoids, which is challenging to implement in practice. Therefore, discrete magnetization (Figure 4b) was used, and its magnetic flux density distribution is shown in Figure 4c. Consequently, a linear correction was applied to correlate the discrete magnetization to the ideal one. Figure 4d illustrates the magnetic flux density before and after correction of a line 3 mm below the lower surface of the magnet (the line from (–5,0,–3) to (5,0,–3)), showing the effectiveness of the correction.

Figure 4e illustrates the usable ranges at different *z* coordinates and magnet radii. The usable range is defined such that the magnetic flux density is corrected by less than 1% of the maximum value from the ideal result. The usable range initially increases and subsequently decreases as *z* decreases. A larger magnet radius results in a larger usable range. However, the radius must be compatible with the supporting columns to avoid interference. Within these constraints, the design parameters were chosen as *t*_*bs*_ = 2 mm, *h*_*bc*_ = 3 mm, and *r*_*bc*_ = 1 mm, for better sensitivity. The value of *r*_*bc*_ can be adjusted according to different application scenarios—larger external loads may require a larger *r*_*bc*_ to extend the measurement range.

The system incorporates three magnets: two positioned on the upper surface of the PCB and one located on the lower surface. Figure 4h shows a schematic of the distribution of magnets and sensors. Figure 4f shows the recovery times under a normal load of 1 N applied to cell (1,1), while those of other cells are shown in Figure S9 (Supporting Information). The times required for cells (1,1), (2,2) and (3,3) to recover to 5% of the deformed state are 0.286, 0.535, and 0.684 s, respectively. Compared to center cells, edge cells recover faster, mainly because they have interactions with fewer neighboring cells.

Figure 4g shows the results of the repeatability test performed by applying a normal load of 0.5 N to the cell (3,3) for 10 000 cycles. The maximum and minimum readings of the SMo tactile skin differ only by 4.07% and 4.88%, respectively, between the first and last 20 cycles. The test demonstrated stable and robust performance in long‐term applications of the SMo tactile skin.

Based on the distribution of magnets and sensors, the sensing area can be divided into 9 zones, as shown in Figure 4h. When a normal force is applied to different zones, different signals (positive or negative) are produced for sensor 2 (S.2) and 3 (S.3), which can be represented by the signal matrix,

(2) $$\text{Signal matrix}\text{(Sm)}\;=\;sign\left[\begin{array}{cc} dS1_{x} & dS1_{y}\\ dS2_{x} & dS2_{y} \end{array}\right]$$

where *dS* denotes the variations in the sensor signals, *x* and *y* specify the direction, and *sign* represents the increase or decrease of the signals (corresponding to positive or negative, respectively). The signal matrix can be used for preliminary localization. Figure 4i shows how magnet M.2 moves and what the signal matrix is when a normal load is applied to the zone Z.1. The signal matrices when loading other zones are shown in Table S1 (Supporting Information).

**Figure 5:**
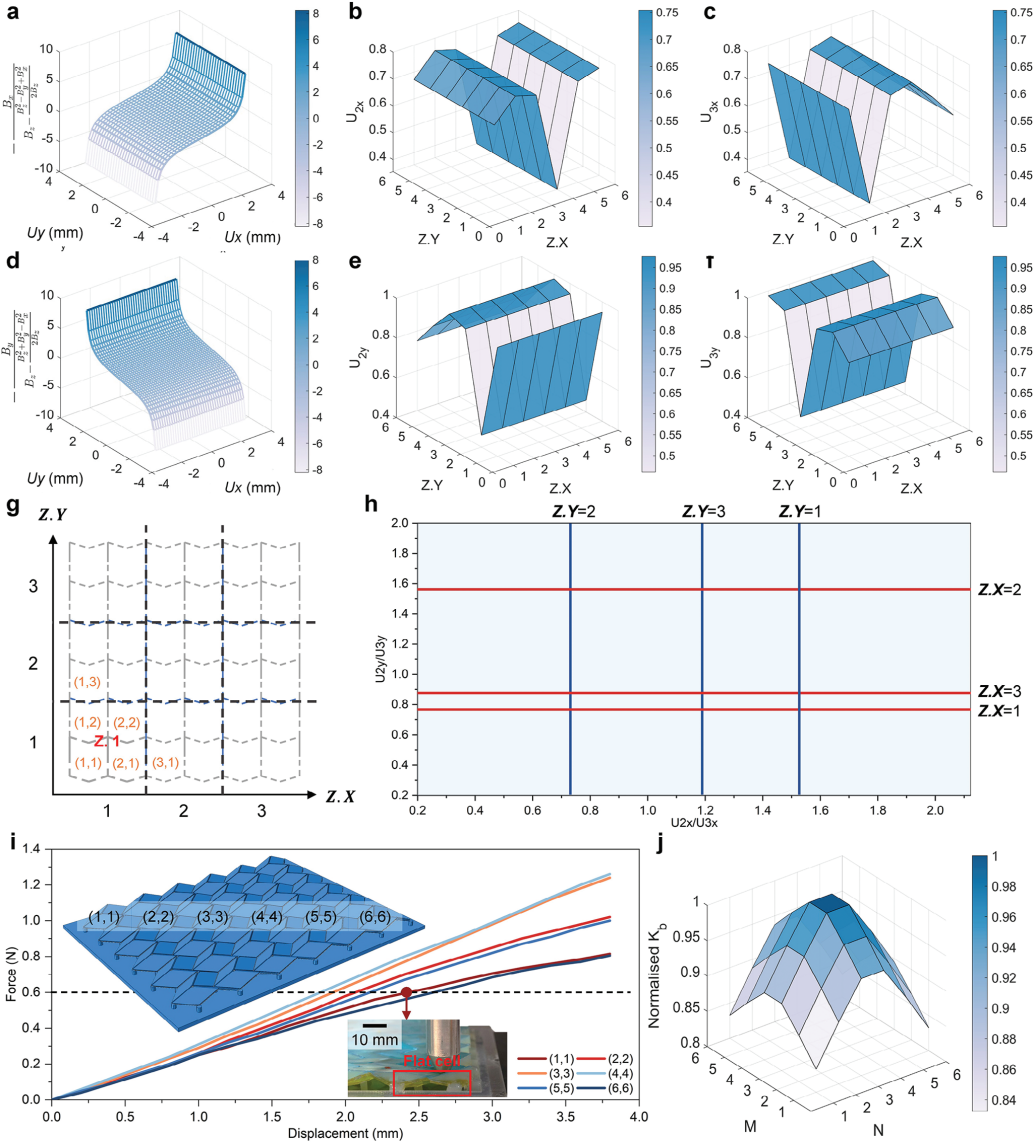
Theoretical model of SMo tactile skin. a) Relationship between $-B_{x}/\left(B_{z}-\left(B_{z}^{2}-B_{y}^{2}+B_{x}^{2}\right)\left(2B_{z}\right)\right)$ and in‐plane displacement. b,c) Theoretical magnet displacement in X direction when a unit displacement *dh* is applied on different cells, for magnets 2 and 3, respectively. d) Relationship between $-B_{y}/\left(B_{z}-\left(B_{z}^{2}+B_{y}^{2}-B_{x}^{2}\right)\left(2B_{z}\right)\right)$ and in‐plane displacement. e,f) Counterparts of (b) and (c) in Y direction. g) Coordinate definition for localization function, where different zones can be described by different values of Z.X and Z.Y, for example, Z.X = 1 and Z.Y = 1 indicates zone1. h) Schematic of localization function. There are different dividing lines for different zones, once the zone is determined, the cell under load can be identified from the displacement relationships *U*_2*x*_/*U*_3*x*_ and *U*_2*y*_/*U*_3*y*_. i) Force‐displacement curve of SMo tactile skin for different cells. Obvious flat of curve can be observed for edge cells under a large load. j) Theoretical normalized stiffness distribution when ν_*a*,*X*_ = ν_*a*,*Y*_ = 0.53.

According to Figure 4h, each zone consists of four cells. Based on the initial localization results using the signal matrix, the actual loaded cell can be further determined by the theoretical model developed. The theoretical model contains the displacement relationships of Miura‐ori bottom vertices, localization function, and the stiffness properties. When a load is applied to the cell (*n*_0_, *m*_0_), the in‐plane displacement of the bottom vertex *U*_*bv*_ of the cell (*n*, *m*) is:

(3) $$U_{bv}\;=\;\left[\begin{array}{c} \sum_{i=1}^{n-n_{0}}\left(\nu_{a,X}^{i}\cdot dl\right)+\frac{dl}{2}\;\sum_{j=1}^{m-m_{0}}\left(\nu_{a,Y}^{j}\cdot dw\right)+\frac{dw}{2} \end{array}\right]{\left[\begin{array}{c} e_{1}\;e_{2} \end{array}\right]}^{T}$$

where ν_*a*,*X*_ and ν_*a*,*Y*_ are the attenuation coefficients, they describe the attenuation of the deformation of other cells when the distance from the loaded cell increases (details are shown in Section S4, Supporting Information), and their values can be obtained from measurements of the deformation characteristics of specific cells and their effects on other cells (called the calibration process, and shown in Section S5, Supporting Information). **e_1_** and **e_2_** are the unit vectors in the X and Y directions, respectively. *dl* and *dw* is the in‐plane geometric size change of the loaded cell (the definitions of *l* and *w* are shown in Figure 3a). The location of external load can be determined through the localization function *Loc* (**Figure** **5**h) using the magnet displacement relationships as:

(4) $$\left[\begin{array}{c} n_{0}\;\;m_{0} \end{array}\right]\;=\;Loc\left(\text{Sm},\;U_{m2}\left(B_{S2}\right),\;U_{m3}\left(B_{S3}\right)\right)$$

where *U*_*m*2_ and *U*_*m*3_ are the displacements of magnets 2 and 3 (M.2 and M.3 in Figure 4), which can be calculated from the magnetic flux density (*B*_*S*2_ and *B*_*S*3_). Then the out‐of‐plane stiffness *K*_*z*_, the estimated shear force *F*_*p*_ and the normal force *F*_*z*_, are:

(5) $$\begin{array}{cc} K_{z}\; & =\;\frac{\partial^{2}\left(E_{C}+E_{b}+E_{P}\right)}{\partial dh^{2}}\\ F_{p}\; & =\;K_{sf}U_{p}\\ F_{z}\; & =\;\left(1+\frac{|F_{p}|}{\nu_{f}}\right)\nu_{s}K_{z}\cdot U_{z}\left(U_{bv},\nu_{a,X},\nu_{a,Y},U_{p}\right) \end{array}$$

*E*_*C*_, *E*_*B*_, and *E*_*P*_ indicate the elastic energies of the creases, the supporting columns, and the panels, respectively. *K*_*sf*_ is the shear stiffness of the shear force sensing layer, while *U*_*p*_ indicates the shear‐induced in‐plane displacement. ν_*f*_ is the folding state correction coefficient compensates for the effects of the shear force, and ν_*s*_ is the stiffness correction coefficient accounts for the stiffness variations resulting from the fabrication process. *U*_*z*_ represents the vertical displacement of the top vertex in the loaded cell, which is a function of *U*_*bv*_, ν_*a*,*X*_, ν_*a*,*Y*_, and *U*_*p*_.

The results from the theoretical model are shown in Figure 5. Figure 5a,d plots the components of the magnetic flux density as functions of the in‐plane displacements. With these relationships, the displacements in the X and Y directions can be solved separately. Figure 5b,c illustrates the X‐direction displacements of two upper magnets when a unit displacement is exerted in different cells, while Figure 5e,f shows the counterparts in the Y direction. Figure 5g,h shows the schematic of the localization function. Combining Figure 5b,c,e–h, the location of the external force can be determined. For example, when the location of an external force is determined in zone Z.1 by the signal matrix, two boundaries: Z.X = 1 and Z.Y = 1 can be selected in Figure 5h. For different values of *U*_2*x*_/*U*_3*x*_ and *U*_2*y*_/*U*_3*y*_, the cell under load can be determined. Details of the localization function for different zones are shown in Figure S5 (Supporting Information). Figure 5i shows the force‐displacement curve of the SMo tactile skin for different cells along the diagonal. Due to an edge effect, edge cells exhibit a notable softening at large displacements, while the stiffness of center cells is relatively stable, e.g. the stiffness of cells (3,3) and (4,4) was attenuated by only 2.2% and 3.2%, respectively. Smaller stiffness leads to a larger displacement under the same load, which further lowers the out‐of‐plane Poisson's ratio and amplifies the softening. It is necessary to determine the values of ν_*a*,*X*_, ν_*a*,*Y*_, and ν_*s*_ to ensure an accurate performance of the model, and Figure 5j shows the calibrated stiffness distribution of the theoretical model.

Based on the theoretical model, the sensing process takes five steps: (1) calculation of the shear‐induced displacement and then the shear force, (2) calculation of the displacements of the two upper magnets and correction with the shear force information, (3) determination of the point of action of the normal force by using the signal matrix and the localization function, (4) evaluation of the displacement of the top vertex, (5) estimation of the magnitude of the normal load using the stiffness properties.

**Figure 6:**
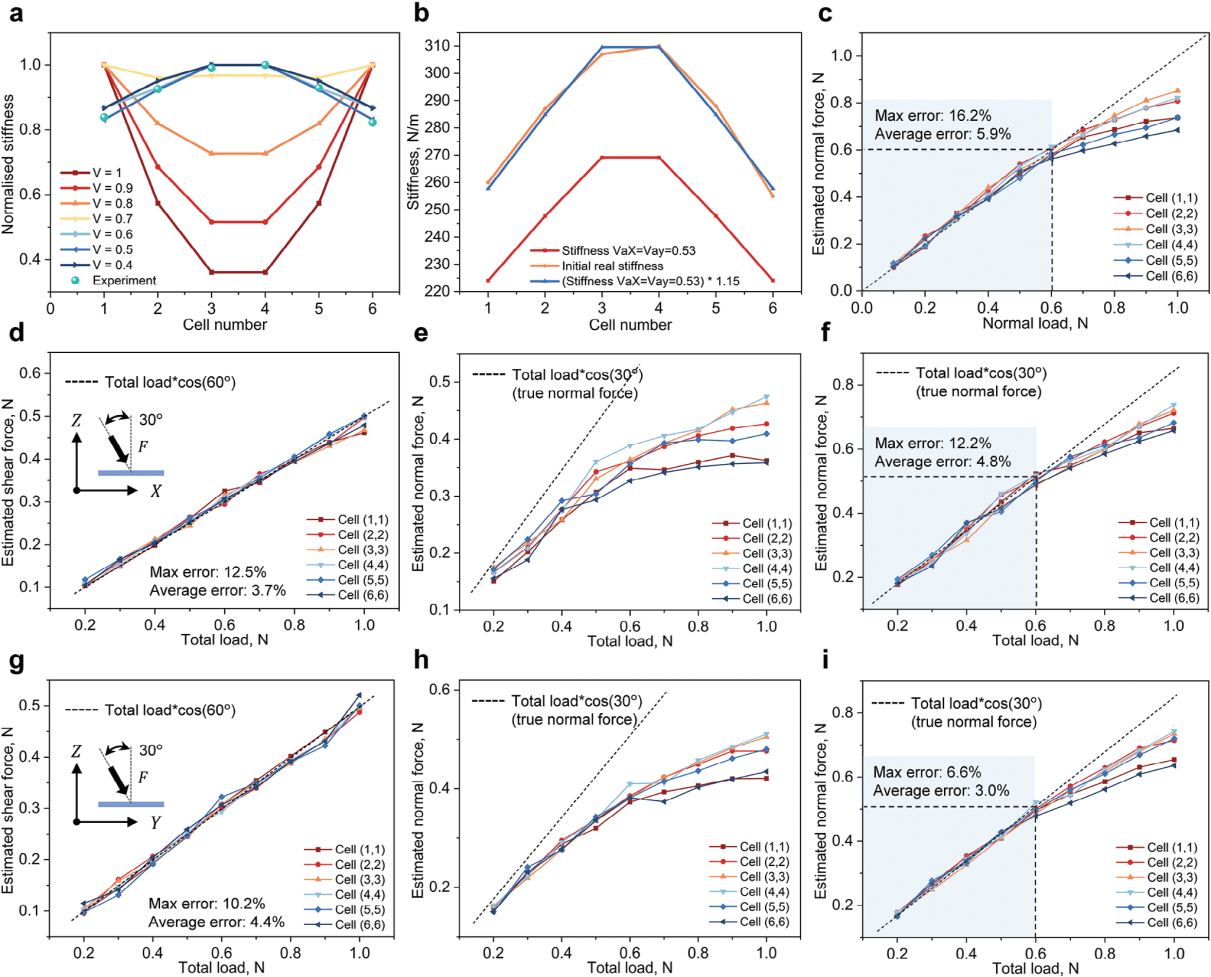
Calibration and performance of the theoretical model. a) Normalized stiffness distribution for different attenuation coefficient (ν_*a*,*X*_ = ν_*a*,*Y*_ = V) and experiment values. Cell number 1 to 6 represents cell (1,1) to (6,6). b) Real stiffness distribution before and after the correction of ν_*s*_. c) Normal force estimation results after calibration. d) Shear force estimation results of load case 1. e) Normal force estimation results of load case 1 before calibration. f) Normal force estimation results of load case 1 after calibration. g) Shear force estimation results of load case 2. h) Normal force estimation results of load case 2 before calibration. i) Normal force estimation results of load case 2 after calibration. Load case 1: both normal force and shear force in X direction are applied. Load case 2: both normal force and shear force in Y direction are applied. For both load cases 1 and 2, the angle between the total forces and the vertical direction is 30°.

The calibration and performance after calibration are demonstrated in **Figure** **6**. Figure 6a,b shows the normalized stiffness distribution for different values of the attenuation coefficients ν_*a*, *X*_ and ν_*a*,*Y*_, and the influence of ν_*s*_ on the true stiffness values, respectively. These two figures can be used for the determination of ν_*a*,*X*_, ν_*a*,*Y*_ and ν_*s*_. Details are shown in Section S5 (Supporting Information). The calibrated values for the skin tested were ν_*a*,*X*_ = ν_*a*,*Y*_ = 0.53 and ν_*s*_ = 1.15.

The normal force estimation performance after calibration is shown in Figure 6c. When only a normal force was applied, the theoretical model performed well for a force less than 0.6 N. An obvious underestimate is seen at a normal load greater than 0.6 N, due to the significant nonlinear behavior of the structure. Therefore, the usable range of the theoretical model for pure normal load sensing is 0–0.6 N.

Figure 6d–f shows the performance of the calibrated model when a mixed load of normal and shear (in the X direction) is applied. For shear force estimation, an accurate estimation in the range of 0–0.5 N (total force 0–1 N) can be achieved, as shown in Figure 6d due to the straightforward sensing mechanism.

The results of the normal force estimation before and after calibration are shown in Figure 6e,f. The calibrated model can obtain an accurate result with a normal force less than 0.52 N (corresponding to a total force of 0.6 N), smaller than in the case of normal force only because of the existence of the shear force. Figure 6g–i shows the performance of the calibrated model when a mixed load of normal and shear (in the Y direction) is applied, which have similar results to those when the shear force is applied in the X direction.

### 2.3 Machine Learning Implementation

The developed theoretical model provides an effective method for localization and force estimation. However, it can only detect forces applied to the top vertices, and the force estimation loses precision under large loads. To further improve localization resolution, increase usable sensing area, and enhance the accuracy of force estimation, the multilayer perceptrons (MLPs)^[^ ^45^, ^46^ ^]^ machine learning method was employed, because of its ability to handle nonlinear problems, as well as its low training cost and straightforward network structure compared to other algorithms such as convolutional neural network (CNN) and recurrent neural network (RNN).

**Figure 7:**
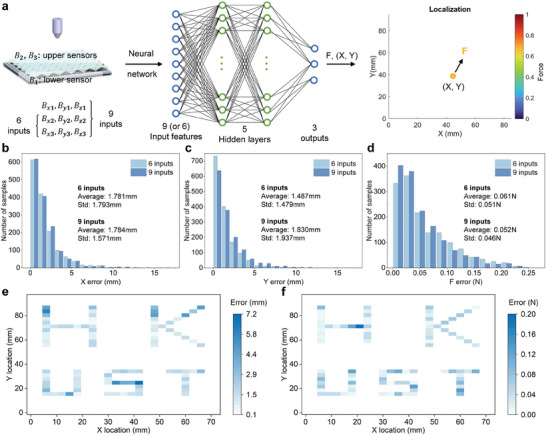
Machine learning implementation and performance. a) Schematic of network. The training data was collected through the testing platform, and then used as the input of the network to train the model, finally the trained model was used for localization and force estimation. b) X localization error of the testing dataset. c) Y localization error of the testing dataset. d) Force estimation error of the testing dataset. e) Average localization error of 5 tests from the demo datasets using the model trained with 9 inputs. f) Average force estimation error of 5 tests from the demo datasets using the model trained with 9 inputs, when a 0.5 N external normal force was applied.

The schematic of MLPs network can be found in **Figure** **7**a. The MLP network features 6 or 9 inputs, 5 hidden layers and 3 outputs. The 5 hidden layers have 64, 256, 1024, 256, and 64 neurons, respectively, and each is followed by a ReLU activation function. A Sigmoid activation function was used at the end of the final output layer since the final outputs were normalized to the range (0,1). The input data consists of the normal force and magnetic flux density variations along the three directions. The 3 outputs denote the force estimation and the X and Y locations, respectively. Before training the network, inputs and outputs were normalized into the range (0,1) to accelerate network convergence.

During data collection to train the network, a series of force‐controlled indentations, up to 0.7 N, were performed across the surface. The load positions span nearly the entire area, ranging from 0 to 72 mm along the X‐axis and from 0 to 91 mm along the Y‐axis, with a uniform spacing of 3 mm. The experiment setup for data capture can be found in Figure S8 (Supporting Information). The total batch size of the experimental dataset is 10 725, divided into a training set (70%), a validation set (15%) and a testing set (15%), and the testing set was used to evaluate the model performances externally.

To investigate the influence of the lower Hall sensor, two different input cases were tested: the first case employed the signals captured from the upper Hall sensors (6 input features), and the second case used the signals from all Hall sensors (9 input features). Figure 7b–d shows the localization errors in the X and Y directions and the force prediction, respectively. The mean error and standard deviation (std) of both test cases are also demonstrated there. The results show that most localization errors along the X and Y axes were less than 5 mm, and most force estimation errors were less than 0.25 N, demonstrating the precision of the MLP network. In addition, the accuracy of localization and force estimation between the 6 and 9 input features was almost the same, mainly because the lower Hall sensor was designed to measure the shear force and is not quite significant in the estimation of the normal force.

We also captured another test sets (demo sets) and each consists of 96 data groups to further demonstrate the potential. This data set was collected with a normal force magnitude of 0.5 N, and the load positions formed the **HKUST** pattern. The test errors for the load positions and force prediction are shown in Figure 7e,f, respectively. In this demo case, the trained model achieved an average localization error of 1.89 mm with a std of 0.8 mm. For the estimation of the normal force, a load of 0.5 N resulted in an average error of 0.04 N (average error of 8%) with an std of 0.02 N. The results illustrate the excellent performance of this trained model for localization and force estimation, and also show the potential of SMo tactile skin to be used as a soft touch panel.

The MLPs network was trained only considering the normal force. In order to further extend the implementation of the machine learning model and measure the normal force and shear force simultaneously, we preprocessed the raw data using the theoretical model, corrected the effects caused by the shear force in the two upper Hall sensors, and then passed the preprocessed signals to the machine learning model trained with 6 input features. The test results are shown in Figure S11 (Supporting Information).

### 2.4 Curved Surface Tessellation and Demo of Large‐area Robotic Skin

**Figure 8:**
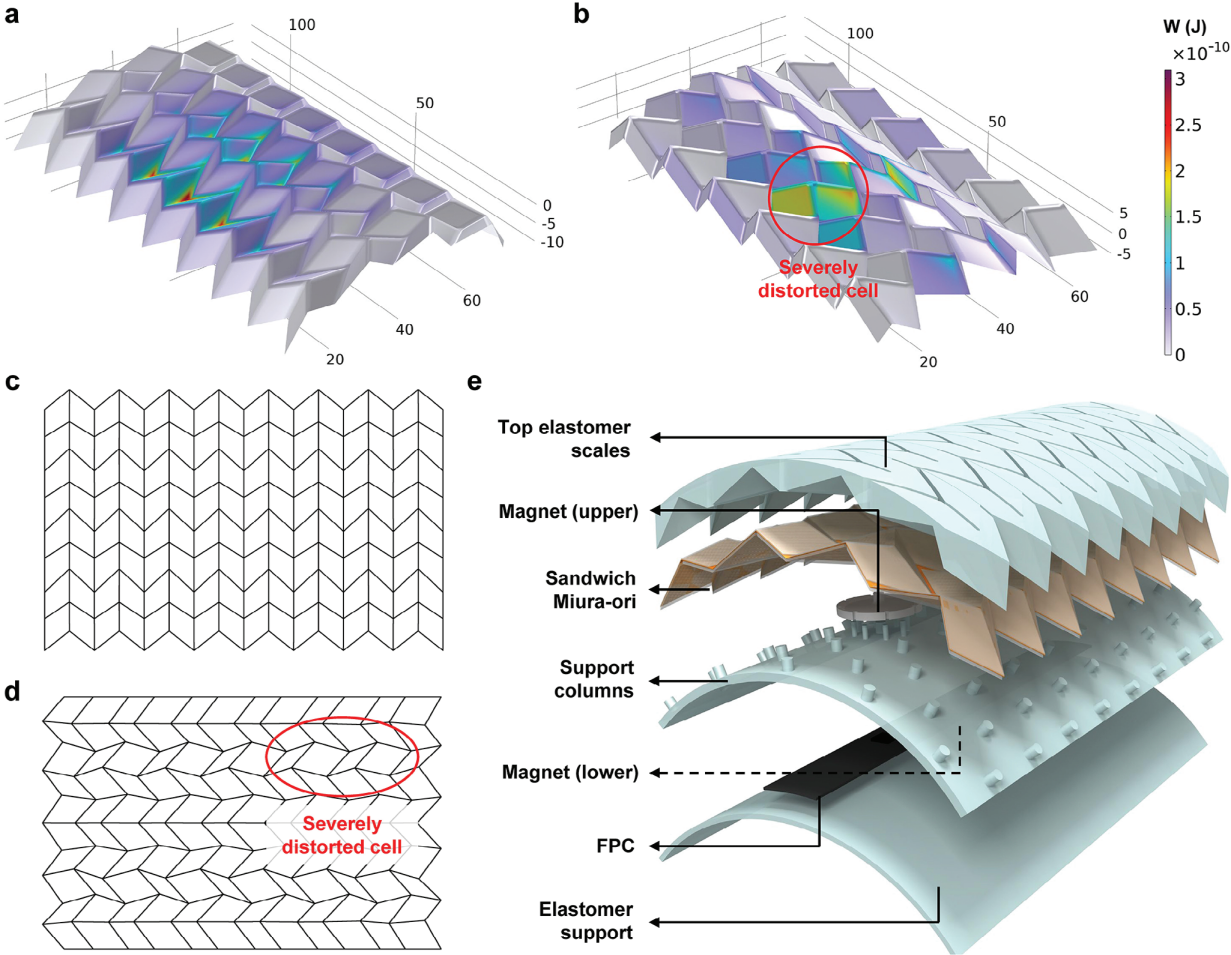
Design of curved SMo tactile skin. a) Strain energy distribution when tessellation along *w* direction. b) Strain energy distribution when tessellation along*l*direction. c) 2D pattern when tessellation along*l*direction. d) 2D pattern when tessellation along *w* direction. Obviously cell distortion can be observed. e) Curved SMo tactile skin structure, which is similar to the planar case.

Miura‐ori structures show potential for conforming to curved surfaces. However, attaching by bending a planar structure introduces residual stress, impairing their folding and unfolding performance. Therefore, an appropriate tessellation algorithm is necessary.^[^ ^37^, ^47^, ^48^ ^]^ In this paper, the curved SMo tactile skin was designed by a previously developed tessellation algorithm^[^ ^42^ ^]^ and then attached to a robotic arm surface for interactions. The tessellation algorithm minimized the total strain energy in the attachment process^[^ ^42^ ^]^:

(6) $$\min_{X(X_{0})}\left(\min_{x(X)}W\left[x\left(X\left(X_{0}\right)\right)\right]\right)$$

where *W* is the strain energy, **X(X_0_)** indicates the in‐plane folding process, and **x(X)** is the attaching process. More details can be found in Section S9 (Supporting Information). The target robotic arm surface is a cylindrical surface with a radius of 52 mm. There were two different tessellation orientations: along the *l* and *w* directions (defined in Figure 3a). The elastic energy distributions for the two directions after tessellation are shown in **Figure** **8**a,b, with their corresponding 2D patterns shown in Figure 8c,d. When tessellation was performed along the *w* direction, all panels exhibit minimal size variation and slight morphological differences from the original state. In contrast, optimization along the *l* direction reduced the maximum strain energy but led to significant variations in the size and shape of the panel. This discrepancy can result in substantial deviations in mechanical properties from the ideal Miura‐ori structure. Therefore, the final optimization direction chosen was the *w* direction. The structure of the curved SMo skin, illustrated in Figure 8e, retains a similar multilayer structure and sensing mechanism as its planar counterpart.

**Figure 9:**
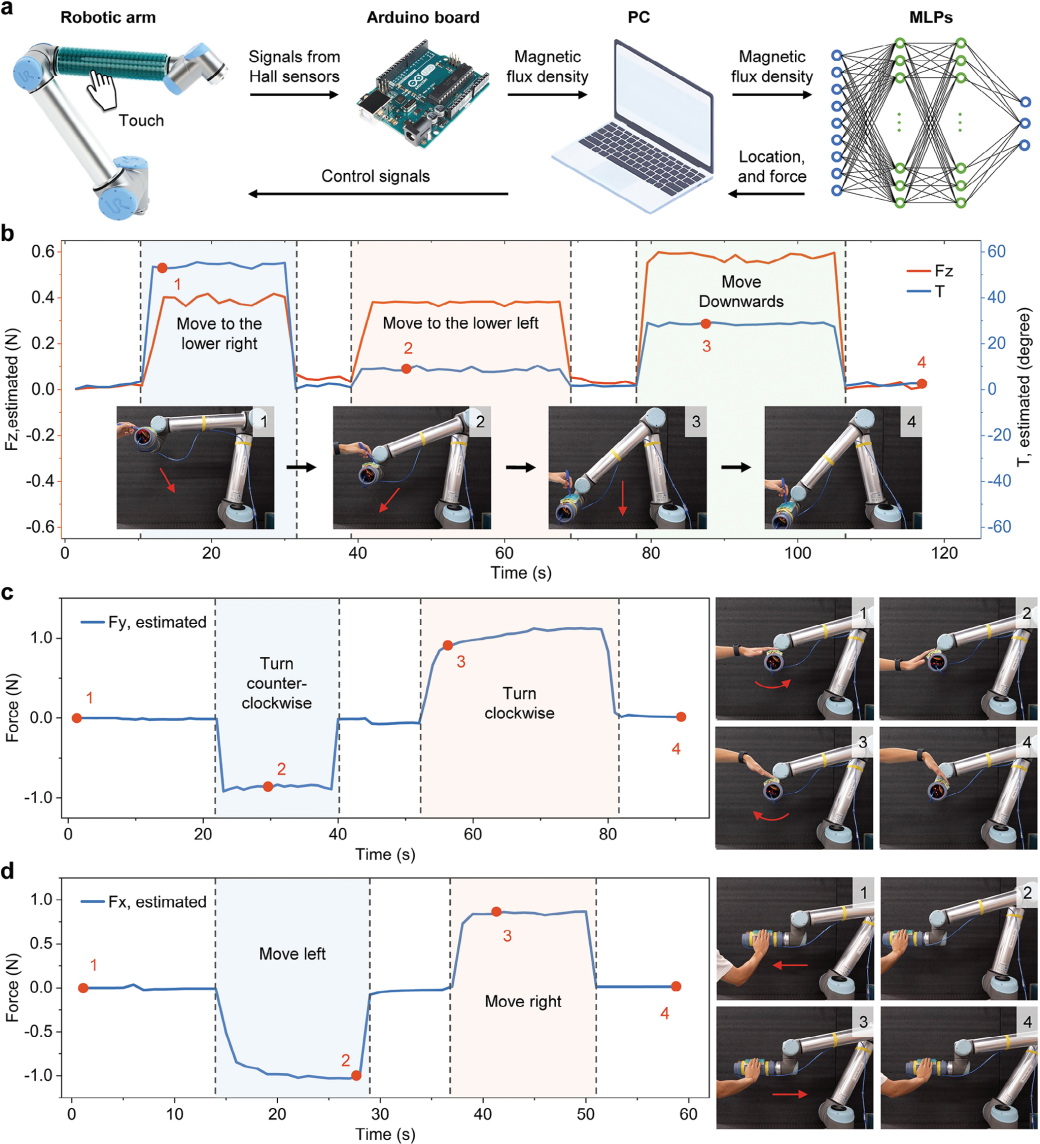
Curved SMo tactile skin for human‐robot interactions. a) Schematic diagram and signal transmission of robotic arm interactions. Hall sensor signals are sent to an Arduino board and then to a laptop after being converted to magnetic flux density values. This magnetic flux density information serves as the input of the machine learning model, allowing for the estimation of the location and magnitude of external loads. Finally, the estimation results are converted into control signals before sent to the robotic arm. b) Robotic arm translation control in direction 1 using normal force. c) Robotic arm rotation control using shear force. d) Robotic arm translation control in direction 2 using shear force.

The curved SMo tactile skin was then fabricated, trained with machine learning algorithm, and installed on a robotic arm surface for interactions, as shown in **Figure** **9**. Figure 9a illustrates the flow of the interaction, and the response time of the entire system was within 0.5 s. Figure 9b demonstrates robotic arm translation control by applying normal force. As normal forces were applied at different locations with different circumferential angles (T), the robotic arm moved along the direction of the external normal load. In subfigures 1, 2, 3, and 4, the robotic arm moved to the lower right, lower left, and downward, since the localization results of circumferential angles were about 50°, 10°, and 30°, respectively. Figure 9c,d shows the rotation and translation control with the shear forces estimated solely based on the theoretical model. The full video of interaction is shown in Movie S1 (Supporting Information). In addition, to illustrate the response of tactile skin to rapid change in external loads, we have developed a visualization interface using MATLAB, and performed another demo of interaction with the robotic arm. In this demo, the location and magnitude of the external force are displayed in the visualization interface in real time, and when the external force is larger than 0.5 N, the control signal is delivered to the robotic arm, and the arm moves in the direction of the external force. Details are shown in Figure S13 and Movie S2 (Supporting Information). This demo shows the ability of SMo tactile skin to respond sensitively to rapidly changing external loads. Apart from direct control, the SMo tactile skin can also be used for collision protection and detection. Its softness, ability to generate a stop signal and to localize contact position upon a collision can minimize accidental damages.

## 3 Conclusion

We developed a novel tactile skin that could achieve large‐area sensing and super‐resolution based on the sandwich Miura‐ori structure. The Miura‐ori structure exhibited a more ideal‐like folding and unfolding behavior due to the optimization of the creases. The multi‐layer design endowed the structure with the capacity to decouple external forces. The developed theoretical model enabled accurate localization and force estimation. Furthermore, the robustness of the theoretical model was enhanced through the incorporation of correction coefficients. The application of machine learning algorithms enabled the extension of the sensing area, resulting in an average localization error of 1.89 mm and an average force estimation error of 8%. Furthermore, a curved SMo tactile skin was developed based on the tessellation algorithm and subsequently employed on a robotic arm for human–robot interactions. This facilitated straightforward control and safe interactions in robotics. This research presented a novel approach to the design and fabrication of large‐area tactile sensors and also addressed the complexity of fabricating and wiring of traditional sensor arrays. The demonstration showcased the device's significant potential in human‐machine interactions and also provided a new solution for future large‐area robotic skins.

## 4 Experimental Section

SMo panels were fabricated from 0.5 mm poly(methyl methacrylate) plate. The middle layer was a 0.05 mm polyimide (PI) film with adhesive on both sides. The magnetic material was a composite mixture of SE 1700 base (DOWSIL), SE 1700 catalyst (DOWSIL), Ecoflex 00‐30 Part B (Smooth‐On Inc.), fumed silica nanoparticles (Aladdin Biochemistry Technology Corp) and NdFeB microparticles (Jianghuai Ciye Corp). The reason for not using Ecoflex Part A + Part B was the excessive heat generated during the mixing process, and the curing would be accelerated, which would be unfavorable for further processing. The upper elastomer scales and supporting columns were made of Ecoflex 00‐30 (Smooth‐On Inc.), and the bottom shear force sensing layer was made of 3D‐printed resin (formlabs, clear V4) and Ecoflex 00‐30.

### Fabrication Process

In the fabrication process, the upper and lower panels were first laser cut into the designed pattern and then bonded with a polyimide (PI) film to form the SMo structure. The performance of layered SMo structure in different temperature is shown in Figure S14 (Supporting Information). The components of the magnetic materials were mixed at 2000 rpm for 2.5 minutes in a planetary mixer, followed by a defoaming process at the same speed for 1.5 min. The magnetic paste was then placed between two aluminum release plates with a designed shape (mold), compressed to a distance of 1 mm, and cured in an oven at 110°C for 1 h. Finally, the material was magnetized in a uniform magnetic field (4.0 T) generated by a pulse coil and assembled manually with a 3D printed magnet holder. The supporting columns, top elastomer scales, and the bottom shear force sensing layer were manufactured using molding techniques. The magnets were then assembled with the 3D printed holder and supporting columns, and the SMo structure was bonded to the supporting columns in bottom vertex areas by applying Ecoflex 00‐30 droplets. Finally, the PCB and bottom shear force sensing layer were assembled, and top elastomer scales were bonded to the upper panels using Silpoxy. The schematic of the fabrication process is shown in Figure S4 (Supporting Information).

### Magnetic Material Characterization

Magnetization loops for five different recipes were tested using MPMS3 (Quantum Design Inc., San Diego, USA), with results presents in Figure S10 (Supporting Information). A higher NdFeB weight ratio corresponds to a higher magnetic moment. However, a higher NdFeB weight ratio also led to a higher viscosity, hindering the degassing process. When the NdFeB weight ratio reached 70%, the magnetic material was too viscous, making the complete defoaming difficult. To ensure quality, the NdFeB weight ratio of 60% was selected. The magnetic flux density in different temperature of the magnet was shown in Figure S15 (Supporting Information), which was tested using a universally applicable lab oven (Memmert UF55Plus, Memmert GmbH, German).

### Circuit Design

The entire circuit consists of three sensors (Magnetometer, MLX90393). The Hall sensors measure the magnetic flux density, then signals were transmitted to an Arduino Uno board via the I2C protocol, before being sent to a laptop for further processing. The I2C communication protocol is a two‐wire interface bus and significantly simplified wiring. The schematic of the circuit design is shown in Figure S3 (Supporting Information).

### Sensor Characterization

External loads were applied using a commercial force gauge (SanLiang Corp.) during the testing, with an indenter installed. The tactile skin was mounted on a platform that can translate in three directions and tilt along two axes, as illustrated in Figure S8a,b (Supporting Information). When applying normal force, the tactile skin was positioned horizontally. For scenarios involving both normal and shear forces, the platform was manually tilted at a specific angle.

### Machine Learning Data Collection

Training data for the machine learning model was collected using a three‐axis mobile platform, as shown in Figure S8c,d (Supporting Information). The sampling intervals were as follows: 1) Planar case: 3 mm sampling intervals were used in both the *X* and *Y* directions, resulting in a total of 825 locations (25×33) for each magnitude of normal load (Overall size: 72 by 96 mm). 2) Curved Surface Case: The *X* interval was maintained at 3 mm, while the circumferential interval was set at 4°, corresponding to an arc length of approximately 3.5 mm (Overall size: 60° by 100 mm). For the magnitude of the normal load, values ranging from 0.1 N to 0.7 N, with increments of 0.05 N, were applied. To ensure that the tactile skin reached a stable state before signal collection, a 2‐s delay was implemented after applying the external load.

### Demonstration Setup

The curved SMo tactile skin was installed on the robotic arm (UR 10, Universal Robots) using double‐sided adhesive. Sensor signals were transmitted to a PC via a USB cable, where localization and force estimation were performed. Based on the location and magnitude of the external load, control signals are generated and sent to the robotic arm through a signal cable.

### Statistical Analysis

Statistical analysis was performed with Microcal Origin Pro (Origin Lab. Corp., Northampton, MA, USA). To ensure data reliability of experimental data, the performance test results in Figure 7e,f were presented as the mean value of 5 different tests.

## Conflict of Interest

The authors declare no conflict of interest.

## Author Contributions

H.Y. conceived the project, H.Y. and W.H. supervised the project. Q.X. designed and fabricated the SMo sample, designed the circuit, and tested the performance. Q.X. and Z.Y. tested the magnet recipe and fabricated the magnets. Q.X. and Z.Y. developed the theoretical model and performed FEA. Q.X. and R.W. built the data collection platform. Z.W. developed machine learning algorithms and trained the data. Q.X. and B.Z. conduct the demos. Y.C., R.J., F.S., and W.H. helped with article writing. All authors contributed to the discussion of the results.

## Supporting information

Supporting Information

Supplemental Movie 1

Supplemental Movie 2

## Data Availability

The data that support the findings of this study are available in the supplementary material of this article.
