# Supplementary material for: Sandwich Miura‐Ori Enabled Large Area, Super Resolution Tactile Skin for Human–Machine Interactions
Source: Adv Sci (Weinh). 2025 Mar 19;12(18):2414580. doi: 10.1002/advs.202414580 (PMC12079333; doi:10.1002/advs.202414580)
Supplement: Supplementary file 1 — Supporting Information [file ADVS-12-2414580-s001.pdf]

ADVANCED SCIENCE
Open Access

Supporting Information

for *Adv. Sci.*, DOI 10.1002/advs.202414580

Sandwich Miura-Ori Enabled Large Area, Super Resolution Tactile Skin for Human–Machine Interactions

Qian Xu, Zhiwei Yang, Zhengjun Wang, Ruoqin Wang, Boyang Zhang, YikKin Cheung, Rui Jiao, Fan Shi, Wei Hong and Hongyu Yu**

Sandwich Miura-Ori Enabled Large Area, Super Resolution Tactile Skin for Human-Machine Interactions - Supplementary Information

Qian Xu[1], Zhiwei Yang[2], Zhengjun Wang[1], Ruoqin Wang[1], Boyang Zhang[1], Yik Kin Cheung[1], Rui Jiao[1], Fan Shi[1], Wei Hong[2,*], and Hongyu Yu[1,*]

[1]The Hong Kong University of Science and Technology, Department of Mechanical and Aerospace Engineering, Hong Kong, 999077, China
[2]Southern University of Science and Technology, Department of Mechanics and Aerospace Engineering, Shenzhen, 518055, China
[*]Corresponding author. Email: hongw@sustech.edu.cn (W.H.); Hongyuyu@ust.hk (H.Y.)

The PDF file includes:

Text S1. Poisson's ratio of Miura-ori structure.
Text S2. Magnetization and correction.
Text S3. Fabrication process.
Text S4. Theoretical model.
Text S5. Calibration process.
Text S6. Magnet material characterization and simulation.
Text S7. Machine learning with preprocessed input.
Text S8. Discussion about multi-point touch.
Text S9. Curved surface tessellation.
Text S10. Demo of Human-Machine Interaction for Rapidly Changing Contact Pressures.
Text S11. Stability testing of sensors in different environments.

Figure S1. Out-of-plane Poisson's ratio of Miura-ori single cell.
Figure S2. SMo-magnets-supporting column connections.
Figure S3. Circuit design.
Figure S4. Fabrication process of SMo tactile skin.
Figure S5. Schematic of localization function for different zones.
Figure S6. Crease and Vertex annotation and Miura-ori single cell geometry definition.
Figure S7. Theoretical model normalized stiffness distribution for different values of $\nu_{a,X}$ and $\nu_{a,Y}$.
Figure S8. Sensor test and data collection platform.
Figure S9. Recovery time when a load of 1N applied on different cells.
Figure S10. Magnet material characterization and soft magnet simulation.
Figure S11. Machine learning performance with preprocessed input when both normal force and shear force are applied.
Figure S12. Performance of curved SMo tactile skin.
Figure S13. Human-machine interaction demo with prescribed external force threshold.
Figure S14. Testing of structural stability at different temperatures.
Figure S15. Influence of temperature on the magnet.
Figure S16. A possible structure to solve the limited sensing area issue without machine learning.

Table S1. Values of signal matrices for different zones.

Other Supplementary Material for this manuscript includes the following:

Movie S1. Demo of human-robotic arm interaction. (.mp4 format).
Movie S2. Response of robotic arm under sudden change of external force. (.mp4 format).

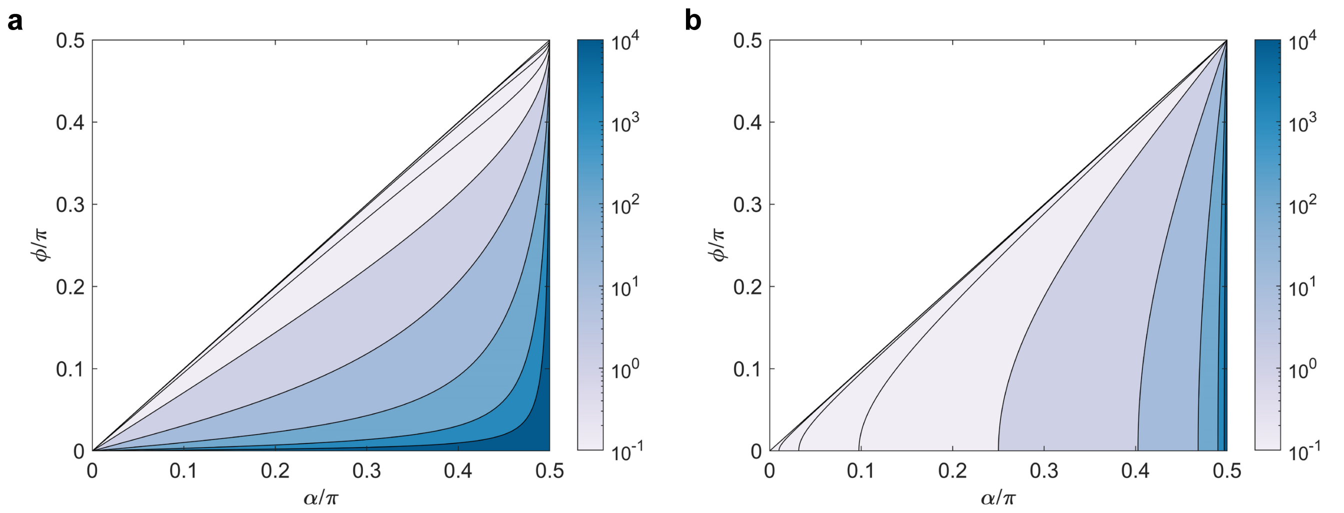

Figure S1. Out-of-plane Poisson's ratio of Miura-ori single cell. (a) ν_{13}. (b) ν_{23}.

Poisson's Ratio of Miura-ori Structure

For a Miura-ori cell, the geometric parameters are delineated in Figure S6. The parameters l, w, and h denote length, width, and height, respectively. The angle α represents the internal angle of the parallelogram panel, while ϕ denotes the folding state angle. a and b are the lengths of the panels. Vectors $\vec{c}_1$ to $\vec{c}_4$ are associated with the four creases. The symbols ① to ⑥ represent the six bottom vertices, and X, Y, Z are the coordinate axis whose origin is located at the top vertex of the cell. Under this definition, the following geometric relationship holds:

$$\begin{aligned} l &= 2b\sin\phi \\ w &= 2a\cos\alpha\sec\phi \\ h &= a\sqrt{\sin^2\alpha - \sin^2\phi}\,\sec\phi \end{aligned} \tag{S1}$$

Then the Poisson's ratios of a single cell are:

$$\begin{aligned} \nu_{12} &= -\frac{dl}{dw}\frac{w}{l} = -\cos^2(\phi) \\ \nu_{13} &= -\frac{dl}{dh}\frac{h}{l} = \cot^2(\phi)\frac{\sin^2(\alpha)-\sin^2(\phi)}{\cos^2(\alpha)} \\ \nu_{23} &= -\frac{dw}{dh}\frac{h}{w} = \frac{\sin^2(\alpha)-\sin^2(\phi)}{\cos^2(\alpha)} \end{aligned} \tag{S2}$$

The values of two out-of-plane Poisson's ratios, ν_{13} and ν_{23} for different values of panel internal angle α and folding state angles ϕ, are shown in Figures S1a and b. These two Poisson's ratios exhibit similar trends as the values of angles α and ϕ vary. For a given value of α, both ν_{13} and ν_{23} decrease as ϕ increases, and this is one of the reasons why the force-displacement curve becomes flat and the stiffness of edge cells decreases dramatically when displacement is large. Similarly, when ϕ is kept constant, both ν_{13} and ν_{23} will increase as α increases.

Magnetization and Correction

A special magnetization is required to induce a special magnetic field, and then achieve force decoupling. Here, centripetal magnetization[1] was adopted, which can be demonstrated by the following expression: Suppose that there is a thin magnet film (or thin magnet plate) with a thickness d, and it is positioned on the XY plane, with its top and bottom surfaces located at $z = d$ and $z = 0$, respectively. If the magnetization direction of the magnet plate is the superposition of two sinusoids in quadrature, which is:

$$
\begin{aligned}
m_x &= m_0 \sin kx \\
m_y &= m_0 \sin ky \\
m_z &= 0
\end{aligned}
\tag{S3}
$$

where k is the wave number, equals $2\pi/\lambda$, and λ is the wave length. Then the magnetic flux density can be derived according to the governing equation of the magnetic field:

$$
\begin{aligned}
B_x &= -Ke^{kz} \sin kx \\
B_y &= -Ke^{kz} \sin ky \\
B_z &= Ke^{kz}(\cos kx + \cos ky)
\end{aligned}
\tag{S4}
$$

where $K = m_0(1 - e^{-kd})/2$. Therefore, the following relationship exists:

$$
\begin{aligned}
\tan kx &= -\frac{B_x}{B_z - \frac{B_z^2 - B_y^2 + B_x^2}{2B_z}} \\
\tan ky &= -\frac{B_y}{B_z - \frac{B_z^2 + B_y^2 - B_x^2}{2B_z}} \\
Ke^{kz} &= \sqrt{(B_z - \frac{B_z^2 + B_y^2 1 B_x^2}{2B_z})^2 + B_y^2}
\end{aligned}
\tag{S5}
$$

Note that in Eq.S5, these three magnetic flux density combinations are solely related to the X, Y, and Z coordinates, thus the displacements in all three directions can be calculated separately based on the magnetic flux density. However, achieving ideal magnetization is challenging due to fabrication challenges. Therefore, discrete magnetization[1], which consists of four magnetized centripetal segments, was adopted to represent the ideal centripetal magnetization. This approach inevitably introduces noticeable errors in the magnetic flux density. To compensate for these differences, corrections must be applied, and a linear correction is sufficient to achieve accurate results within a specific range near the center of magnet. The linear corrected magnetic field can be expressed with the following expression:

$$
B_c = k_c \cdot B + c_c \tag{S6}
$$

where k_c is the linear correction coefficient, c_c represents the translational value, B denotes the original magnetic flux density of centripetal magnetization and B_c is the corrected magnetic flux density. After applying this correction, the magnetic flux density of the discrete magnetization closely approximates that of the ideal magnetization within a specific range near the center, referred to as the "usable range".

Structure Connections

The magnets were first attached to a holder, and then the holder was connected to the bottom support columns through notches on the edge of the holder, which is shown in Figure S2a. The Miura-ori structure was connected to the bottom support columns in the areas of the bottom vertex, as the bottom support columns are designed to be perfectly aligned with the SMo bottom vertices, which is shown in Figure S2b. In addition, there is also a column on the upper surface of the magnet holder to connect with the Miura-ori structure. This column ensures that the magnets can move in accordance with the unfolding of the Miura-ori structure.

Circuit Design

The PCB circuit design for the planar SMo tactile skin is illustrated in Figure S3. The design of the flexible printed circuit (FPC) for the curved SMo tactile skin follows a similar approach to that of the planar PCB, and the main difference is that there are four Hall sensors (MLX90393) on FPC, three on the upper surface and one on the lower surface, where the total number of Hall sensors was 3 on planar PCB.

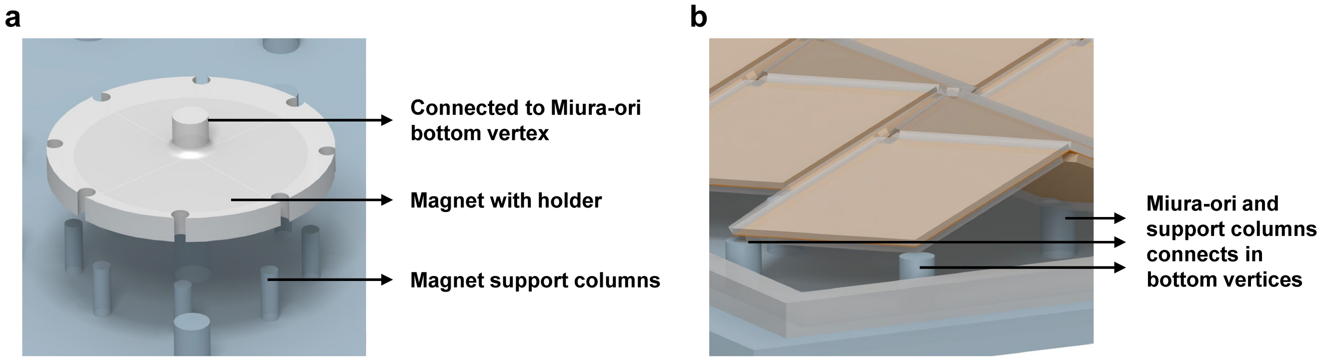

Figure S2. **SMo-magnets-support column connections.** **(a)** Connection between magnet and bottom support columns. **(b)** Connection between SMo and bottom support columns.

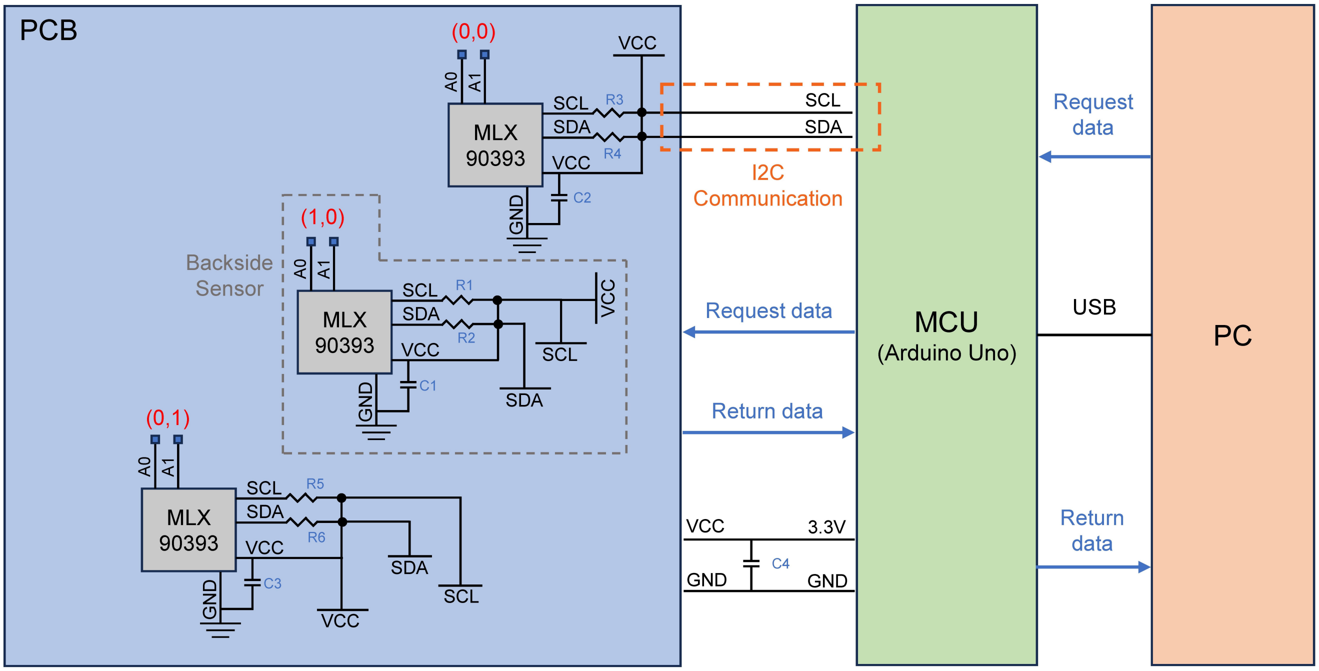

Figure S3. **Circuit design.**

Fabrication Process

The fabrication process is described in Figure S4, with the following steps: (1,1), (1,2), and (1,3) involve the fabrication of the SMo structure. Crease-optimized Miura-ori patterns for the upper and lower surfaces were cut from a thin acrylic plate by laser cutting, and then the patterns were bonded to a thin PI film with double-layer adhesives, and the redundant portion was cut and the vertex holed to form a planar SMo structure. With the help of the folding mold, all the creases can be folded in the prescribed folding directions. (2,1), (2,2), and (2,3) detail the fabrication of magnets with the prescribed magnetization. At first, magnetic mud was placed into a mold with designed shape, and then aluminum plates were used to remove excess mud. After heating in the oven at 110 °C for 1 hour, the magnet mud cured and then was magnetized in a designed direction by a magnetizing coil. Finally, four sector magnets are assembled inside the holder to form a complete magnet. (3,1) and (3,2) cover the fabrication of support columns. Ecoflex-0030 was injected into the mold with a syringe, and after curing and demolding, the fabrication of support columns is finished. (4) shows the assembly of the SMo structure, magnets, and support columns. SMo stricture was aligned with bottom support columns in bottom vertices, and the folding state was restricted by a frame to align with columns. Then Ecoflex-0030 was dripped in the bottom vertices areas to connect the SMo and bottom support columns. (5), (6), and (7) relate to the fabrication of the bottom shear sensing layer, the PCB, and the top elastomer scales, respectively. The shear force sensing layer was made of a 3D printed base, with some bonded thin elastomer support to support

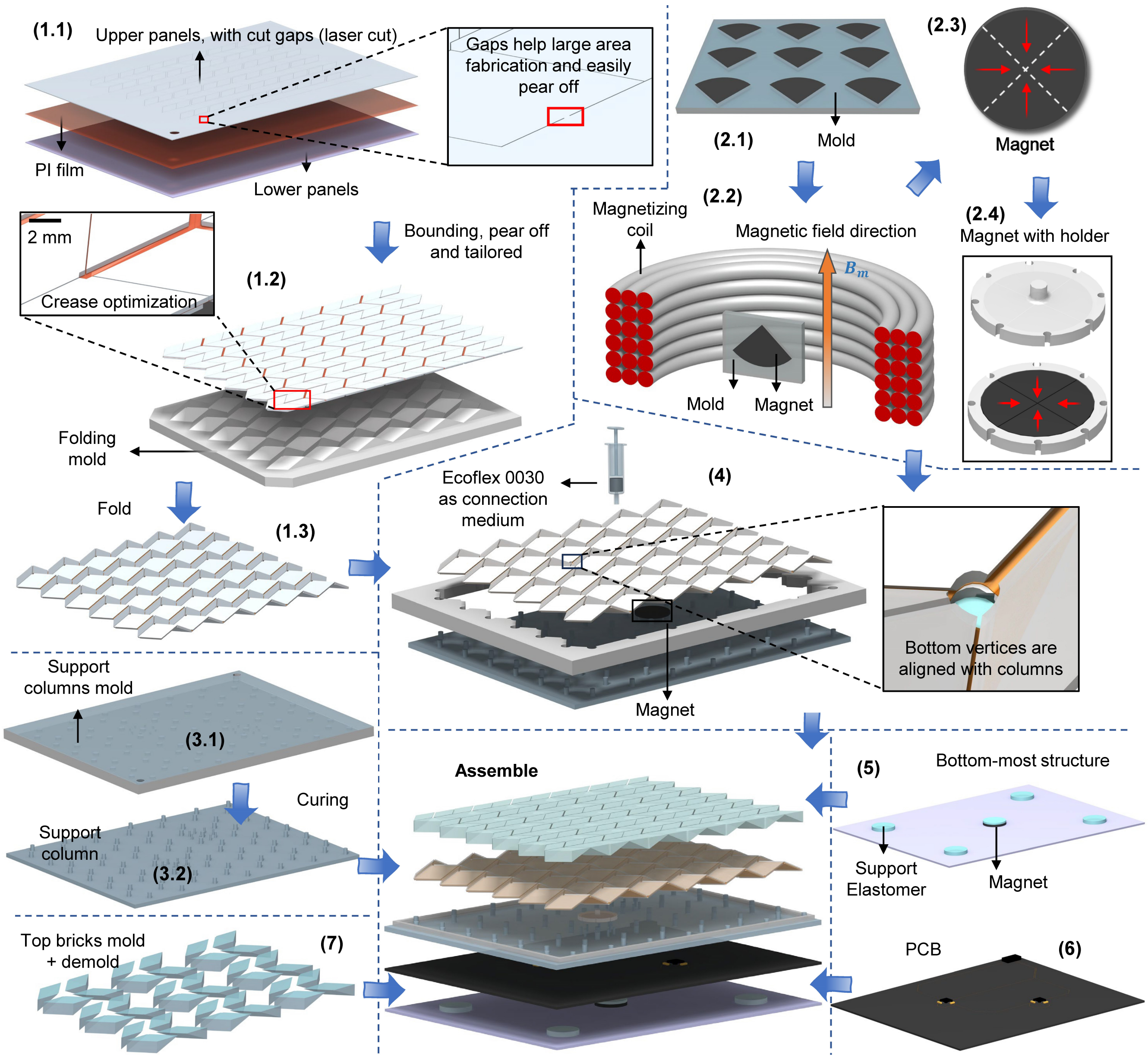

Figure S4. Fabrication process of SMo tactile skin. (1,1), (1,2), and (1,3), fabrication of SMo structure. (2,1), (2,2), and (2,3), fabrication of magnets with prescribed magnetization. (3,1) and (3,2), fabrication of support columns. (4), assembly of the SMo structure, magnets, and support columns. (5), (6), and (7), fabrication of the bottom-most support, PCB, and top elastomer scales.

the PCB, and also to ensure there will be a deformation when shear load is applied. The top elastomer scales were made of Ecoflex-0030 by molding, which is similar to the bottom support columns. Finally, all components are assembled together to form the SMo tactile skin. This fabrication process was simple, low-cost, and well suited for large-area manufacturing.

Theoretical Model

The developed model is a simplified representation with the following assumptions: 1) The entire structure operates within a linear deformation range, indicating that the relationship between stress and strain is linear. 2) At the point (top vertex of a cell) where a normal load is applied, displacement occurs exclusively in the Z direction. 3) Each cell functions as the smallest unit, maintaining a consistent folding state within each unit. (4) All loads are applied in the top vertices of Miura-ori cells.

The first part of the theoretical mechanical model focuses on the deformation and displacement of the bottom vertices of the

Miura-ori structure (which is also the displacement of the bottom support columns). Under the previously stated assumptions, and based on the geometric relationship given in Equation (S1) together with the definition of Poisson's ratio, for a single Miura-ori cell, if the height changes by dh (with positive values indicating an increase and negative values indicating a decrease), the corresponding changes in the in-plane dimensions are as follows:

$$\begin{aligned} dl &= -\frac{dh}{h} l \cdot \nu_{13} \\ dw &= -\frac{dh}{h} w \cdot \nu_{23} \end{aligned} \tag{S7}$$

For a single cell, there are 6 bottom vertices, labeled ① to ⑥, as shown in Figure S6. Assuming that the origin of the local coordinate system is located at the top center vertex, when the top vertex moves dh, the displacements of the bottom vertices in the local coordinate system are: $d_{2x,local} = d_{5x,local} = 0$, $d_{2y,local} = -d_{5y,local} = dw/2$, $d_{1x,local} = d_{6x,local} = -d_{3x,local} = -d_{4x,local} = dl/2$, for the displacements of the bottom vertices ①, ③, ④, and ⑥ in the Y direction, they are:

$$\begin{aligned} d_{1y,local} &= d_{3y,local} = [\frac{w+dw}{2} + bcos(\phi + d\phi)] - [\frac{w}{2} + bcos(\phi)] \\ d_{4y,local} &= d_{6y,local} = [-\frac{w+dw}{2} + bcos(\phi + d\phi)] - [-\frac{w}{2} + bcos(\phi)] \end{aligned} \tag{S8}$$

So, the local displacement can be written as

$$\begin{aligned} u_{1,local} &= \frac{dl}{2} \cdot \mathbf{e}_1 + [(\frac{w+dw}{2} + bcos(\phi + d\phi)) - (\frac{w}{2} + bcos(\phi))] \cdot \mathbf{e}_2 \\ u_{2,local} &= 0 \cdot \mathbf{e}_1 + \frac{dw}{2} \cdot \mathbf{e}_2 \\ u_{3,local} &= -\frac{dl}{2} \cdot \mathbf{e}_1 + [(\frac{w+dw}{2} + bcos(\phi + d\phi)) - (\frac{w}{2} + bcos(\phi))] \cdot \mathbf{e}_2 \\ u_{4,local} &= -\frac{dl}{2} \cdot \mathbf{e}_1 + [(-\frac{w+dw}{2} + bcos(\phi + d\phi)) - (-\frac{w}{2} + bcos(\phi))] \cdot \mathbf{e}_2 \\ u_{5,local} &= 0 \cdot \mathbf{e}_1 + -\frac{dw}{2} \cdot \mathbf{e}_2 \\ u_{6,local} &= \frac{dl}{2} \cdot \mathbf{e}_1 + [(-\frac{w+dw}{2} + bcos(\phi + d\phi)) - (-\frac{w}{2} + bcos(\phi))] \cdot \mathbf{e}_2 \end{aligned} \tag{S9}$$

Note that ϕ, l and w can be expressed by h, therefore $d\phi$, dl and dw can be expressed by dh. Considering the global scenario where an external load is applied to the cell (n_0, m_0). It is evident that, under rigid-foldable conditions, the in-plane displacement of the top vertex of the cell located at (n,m) is $(n-n_0)dl \cdot \mathbf{e}_1$ and $(m-m_0)dw \cdot \mathbf{e}_2$, in the X and Y directions, respectively. Taking into account the attenuation coefficient $\nu_{a,X}$ and $\nu_{a,Y}$, which is explained again here for convenience: if the change in in-plane size of cell (m,n) under the rigid-foldable assumption is (dl_0, dw_0), and the actual change in in-plane size is (dl, dw), with an external load applied at (n_0, m_0), the relationship can be expressed as follows:

$$\begin{aligned} dl &= dl_0 \cdot \nu_{a,X}^{|n-n_0|} \\ dw &= dw_0 \cdot \nu_{a,Y}^{|m-m_0|} \end{aligned} \tag{S10}$$

Therefore, the total in-plane displacement of arbitrary bottom vertex is the superposition of local displacement and the displacement of local coordinate:

$$
\begin{aligned}
\mathbf{u_1} &= \begin{bmatrix} (v_{a,X}^1+v_{a,X}^2+\ldots+v_{a,X}^{|n-n_0|})dl+\frac{1}{2}dl \\ (v_{a,Y}^1+v_{a,Y}^2+\ldots+v_{a,Y}^{|m-m_0|})dw+[\frac{w+dw}{2}+bcos(\phi+d\phi)]-[\frac{w}{2}+bcos(\phi)] \end{bmatrix}^{\mathrm{T}} \\
\mathbf{u_2} &= \begin{bmatrix} (v_{a,X}^1+v_{a,X}^2+\ldots+\frac{1}{2}v_{a,X}^{|n-n_0|})dl+\frac{1}{2}dl \\ (v_{a,Y}^1+v_{a,Y}^2+\ldots+v_{a,Y}^{|m-m_0|})dw+\frac{1}{2}dw \end{bmatrix}^{\mathrm{T}} \\
\mathbf{u_3} &= \begin{bmatrix} (v_{a,X}^1+v_{a,X}^2+\ldots+v_{a,X}^{|n-n_0-1|})dl+\frac{1}{2}dl \\ (v_{a,Y}^1+v_{a,Y}^2+\ldots+v_{a,Y}^{|m-m_0|})dw+[\frac{w+dw}{2}+bcos(\phi+d\phi)]-[\frac{w}{2}+bcos(\phi)] \end{bmatrix}^{\mathrm{T}} \\
\mathbf{u_4} &= \begin{bmatrix} (v_{a,X}^1+v_{a,X}^2+\ldots+v_{a,X}^{|n-n_0-1|})dl+\frac{1}{2}dl \\ (v_{a,Y}^1+v_{a,Y}^2+\ldots+v_{a,Y}^{|m-m_0-1|})dw+[\frac{w+dw}{2}+bcos(\phi+d\phi)]-[\frac{w}{2}+bcos(\phi)] \end{bmatrix}^{\mathrm{T}} \\
\mathbf{u_5} &= \begin{bmatrix} (v_{a,X}^1+v_{a,X}^2+\ldots+\frac{1}{2}v_{a,X}^{|n-n_0|})dl+\frac{1}{2}dl \\ (v_{a,Y}^1+v_{a,Y}^2+\ldots+v_{a,Y}^{|m-m_0-1|})dw+\frac{1}{2}dw \end{bmatrix}^{\mathrm{T}} \\
\mathbf{u_6} &= \begin{bmatrix} (v_{a,X}^1+v_{a,X}^2+\ldots+v_{a,X}^{|n-n_0|})dl+\frac{1}{2}dl \\ (v_{a,Y}^1+v_{a,Y}^2+\ldots+v_{a,Y}^{|m-m_0-1|})dw+[\frac{w+dw}{2}+bcos(\phi+d\phi)]-[\frac{w}{2}+bcos(\phi)] \end{bmatrix}^{\mathrm{T}}
\end{aligned}
\tag{S11}
$$

Note that in Eq.(S11), dl and dw is the change in the size of the cell under load. According to the derived expression, the localization of the external load (only normal force is applied) can be conducted:

$$
\begin{bmatrix} x_F & y_F \end{bmatrix} = Loc\left(\mathrm{Sm},\ U_{m2}(B_{S2}),\ U_{m3}(B_{S3})\right) \tag{S12}
$$

where $\begin{bmatrix} x_F & y_F \end{bmatrix}$ is the location of external load, Loc is the localization function. The schematic of the localization function for different zones (from zone 1 to zone 9) are shown in Figure S5. In the localization process, the signal matrix determines which zone the force is applied to, and then the localization function identifies which cell inside the zone is under load. For example, if all the elements of Sm are negative, then we can know that the external load is exerted on zone 1, which is the left bottom corner composed of cells (1,1), (2,1), (1,2) and (2,2). Then according to the theoretical model, if a unit displacement dh is applied on top vertex of cell (1,1), the ratio of the displacement of the magnets 2 and 3 in the X direction should be 0.6721/0.7313, and that in Y direction should be 0.9476/0.8709, if the displacement ratio derived from magnetic flux density change meets these two ratios, then the external load can be identified as applied on cell (1,1). In practice, the ratio of magnet displacement will not strictly satisfy the theoretical ratio, so here we take the average value of the ratio of magnet displacement of different cells as the dividing line for judgment, such as zone 1, as shown in Figure S5g, for U_{2x}/U_{3x}, the dividing line for cell (1,1) and (2,1) is U_{2x}/U_{3x} = 1.51. Sm is the signal matrix, U_{m2} and U_{m3} are the displacements of the magnets on the upper surface, which can be calculated from their corresponding magnetic field variations.

The second part is the calculation of elastic energy. The total elastic energy is distributed among three categories: crease bending energy, support column deformation energy, and panel in-plane bending energy. To calculate the elastic energy and stiffness, fundamental geometric relationships need to be derived first. According to the geometric relationships described in Eq.(S1), the size of a single cell can be expressed in terms of the dihedral angles ϕ_1 (or ϕ_2), where ϕ_1 and ϕ_2 are the dihedral angles corresponding to the vectors $\vec{c_1}$ and $\vec{c_2}$ shown in Figure S6. Consequently, w, l, and h can be expressed as:

$$
\begin{aligned}
w &= \frac{2a\cos\alpha}{(1-\sin^2\alpha\sin^2\frac{\phi_1}{2})^{\frac{1}{2}}} \\
l &= 2b\sin\alpha\sin\frac{\phi_1}{2} \\
h &= (a^2-(\frac{w}{2})^2)^{\frac{1}{2}} = \frac{a\sin\alpha\cos\frac{\phi_1}{2}}{(1-\sin^2\alpha\sin^2\frac{\phi_1}{2})^{\frac{1}{2}}}
\end{aligned}
\tag{S13}
$$

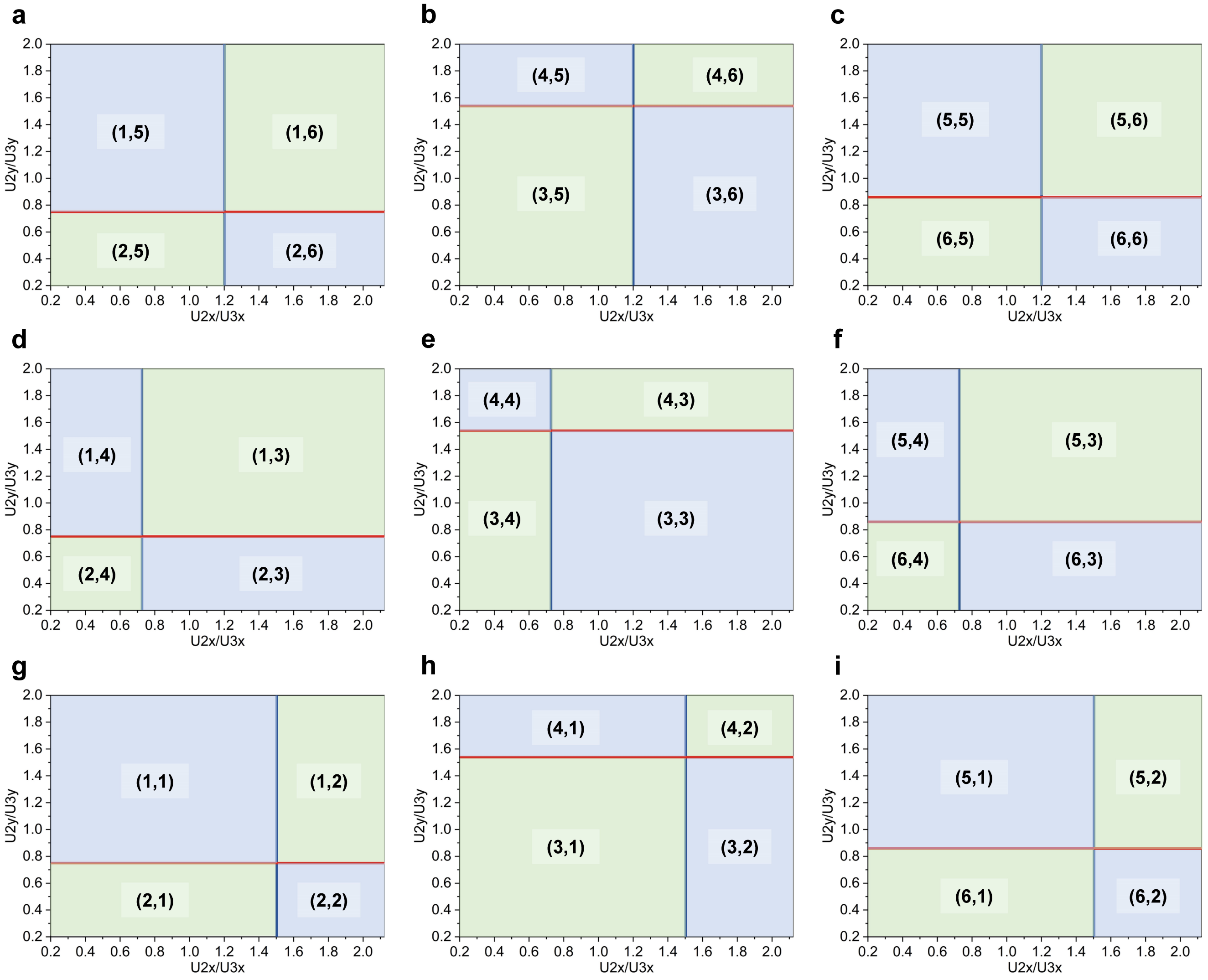

Figure S5. Schematic of localization function for different zones. **(a)** For zone 7. **(b)** For zone 8. **(c)** For zone 9. **(d)** For zone 4. **(e)** For zone 5. **(f)** For zone 6. **(g)** For zone 1. **(h)** For zone 2. **(i)** For zone 3.

Finally, the relationship between the two angles ϕ_1 and ϕ_2 can be derived:

$$a\sin\alpha\cos\frac{\phi_2}{2} = h = \frac{a\sin\alpha\cos\frac{\phi_1}{2}}{(1-\sin^2\alpha\sin^2\frac{\phi_1}{2})^{\frac{1}{2}}} \tag{S14}$$

which indicates ϕ_2 can be expressed by ϕ_1, and vice versa. It can also be seen as a geometric constraint for rigid-foldable Miura-ori.

$$\begin{aligned}\phi_2 &= 2\arcsin\left(\frac{\cos\alpha\sin\frac{\phi_1}{2}}{(1-\sin^2\alpha\sin^2\frac{\phi_1}{2})^{\frac{1}{2}}}\right)\\ \phi_1 &= 2\arcsin\left(\frac{\sin\frac{\phi_2}{2}}{(\cos^2\alpha+\sin^2\alpha\sin^2\frac{\phi_2}{2})^{\frac{1}{2}}}\right)\end{aligned} \tag{S15}$$

The elastic energy of creases can be expressed as the square of the dihedral angles[2]:

$$E_{cs} = 2k_1 a(d\phi_1)^2 + 2k_2 b(d\phi_2)^2 \tag{S16}$$

where E_{cs} denotes the crease energy associated with the four top creases connected to the top vertex of a single cell, as illustrated in the right-hand side of Figure S6. The parameters k_1 and k_2 represent the stiffness of the straight crease, with $k_1 = k_2 = E_c I_Z / a$ (or $E_c I_Z / b$), where E_c is the elastic modulus of the crease material, I_Z is the area moment of inertia per unit length, given by $t_c^3/12$ and t_c is the crease thickness. The parameters a and b denote the widths of the creases.

Under the previous assumption, the external force can be calculated as the first-order derivative of elastic energy with respect to the corresponding displacement, while the stiffness is given by the second-order derivative of elastic energy with respect to the displacement. In Eq.(S16), $d\phi_1$ can be expressed in terms of $d\phi_2$ according to Eq.(S15), and $d\phi_2$ can be expressed in terms of dh according to Equation (S14). Therefore, the crease energy can be formulated as a function of the vertical displacement dh:

$$E_{cs} = f_{cs}(d\phi_2(dh), d\phi_1(dh)) = f_{cs}(dh) \tag{S17}$$

where:

$$\begin{aligned} d\phi_2(dh) &= -\frac{2}{a \sin\alpha \sin\frac{\phi_2}{2}} dh \\ d\phi_1(dh) &= \frac{2\sqrt{1-\sin^2(\alpha)\sin^2\left(\frac{\phi_1}{2}\right)}\left(\sin^2(\alpha)\sin^2\left(\frac{\phi_1}{2}\right)-1\right)}{a\cos^2(\alpha)\sin(\alpha)\sin\left(\frac{\phi_1}{2}\right)} dh \end{aligned} \tag{S18}$$

Eq.(S16) considers only the four creases of single cell, and for different cells, the folding states are different, resulting in a different value of dh. In the previous analysis, we have introduced the attenuation coefficient, which can be used to calculate the size change of different cells. Combining the definition of Poisson's ratio in Eq.(S7), dh can be calculated from dl or dw for different cells. Therefore, when the size of the cell is changed by dl, the changes in the size of all other cells can be calculated according to the attenuation coefficient, thus dh for different cells, and then the angles ϕ_1 and ϕ_2 can also be calculated, and finally the crease energy can be calculated for different cells. In the global case, suppose there are $N \times M$ cells in total, and suppose that the bottom connected to a cell has the same folding state as that connected with top creases, while the crease annotations are shown in Figure S6. Therefore, the total crease energy E_C can be calculated using the following:

$$E_C = \sum_{n=1}^{N}\sum_{m=1}^{M} f_{cs,(n,m)}(dh) + \sum_{n=1}^{N-1}\sum_{n=1}^{M-1} f_{cs,(n,m)}(dh) \tag{S19}$$

where $f_{cs,(n,m)}(dh)$ represents the elastic energy of the four creases leading to the top vertex of the cell located at (n,m). The latter term refers to the energy associated with the bottom creases. Consequently, the crease stiffness K_C is the second-order derivative of the crease energy with respect to the displacement dh of the cell under load:

$$K_C = \frac{\partial^2 E_C}{\partial dh^2} \tag{S20}$$

Next, for the energy associated with the bottom support columns, all the columns are simplified to be linear springs that deform in the X-Y plane, and with a constant in-plane stiffness k_{bc}, thus the following expression can be established,

$$F_{bc} = S \cdot (G_b \cdot \gamma) = S \cdot G_b \cdot \frac{du}{h_{bc}} = k_{bc} \cdot du \tag{S21}$$

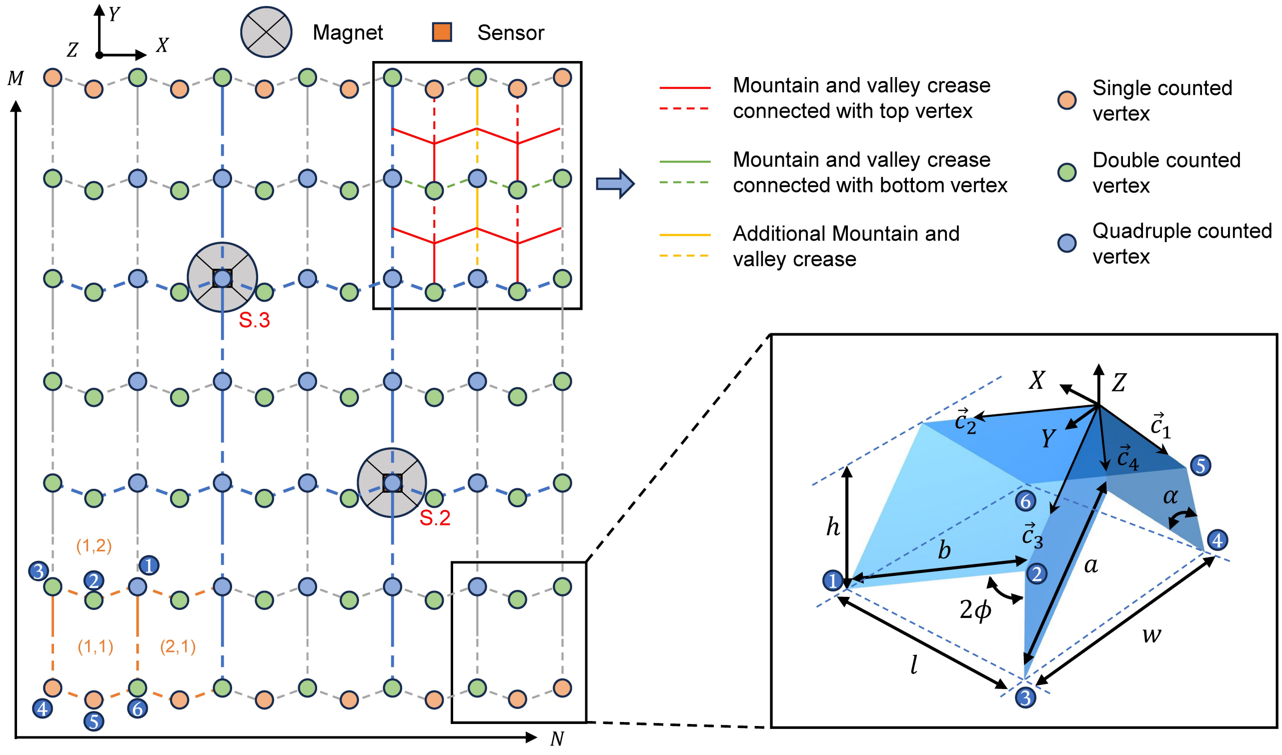

Figure S6. Crease and Vertex annotation and Miura-ori single cell geometry definition. Solid line represents mountain creases, and the dashed line is the valley creases. Different kinds of bottom support columns are annotated by circles of different colors.

where F_{bc} is the in-plane reaction force of support columns, S is the cross-section area of the bottom support columns, G_b is the shear modulus of bottom support column material, γ is the shear strain, du is the in-plane displacement, and h_{bc} is the height of the bottom support columns. Therefore, the elastic energy of six bottom support columns corresponding to a single cell is:

$$E_{bs} = \frac{1}{2} k_{bc} \sum_{i=1}^{6} \mathbf{u_i}^2 \tag{S22}$$

Note that in Equation (S22), $\mathbf{u_i}$ represents the total displacement (in the global coordinate system), with the attenuation coefficients already incorporated. Therefore, the total elastic energy of bottom support columns is the sum of elastic energy for all columns,

$$E_B = \sum_{m=1}^{M} \sum_{n=1}^{N} E_{bs,(n,m)} - \sum E_{bc,\ double} - 3 \sum E_{bc,\ quadruple} \tag{S23}$$

$\sum E_{bc,\ double}$ is the elastic energy of the columns counted twice and $\sum E_{bc,\ quadruple}$ is that counted four times since neighboring cells will share some same support columns, which is shown in Figure S6, and the geometry definitions of a Miura-ori single cell are also shown here again for convenience. Then the stiffness of the bottom support columns is:

$$K_B = \frac{\partial^2 E_B}{\partial dh^2} \tag{S24}$$

The panel bending and in-plane stretching energy E_p as well as its induced stiffness K_p can also be calculated according to the different folding states of the neighboring cells. Then the total stiffness is:

$$K_z = K_C + K_B + K_p \tag{S25}$$

In contrast to the elastic energy of the crease and the elastic energy of the bottom support columns, the bending and stretching energy of the panel E_p and its associated stiffness K_p are relatively minor and can be considered negligible. Consequently, the total stiffness can be approximated by the sum of the stiffness contributions from the crease and the bottom support. Moreover, the crease thickness is quite small, thus its bending energy is substantially less than the elastic energy of the bottom support columns. Therefore, it may also be ignored if necessary.

The initial step in sensing and force estimation is the estimation of the shear force, as it is not affected by the normal load. The hall sensor located on the lower surface of the PCB is responsible for measuring the shear load. The principle for estimating the in-plane shear force F_p is straightforward:

$$F_p = K_{sf} U_p \tag{S26}$$

where K_{sf} represents the shear stiffness of the bottom shear force sensing layer, which can be easily calculated in a similar way to the bottom support columns. The term U_p denotes the in-plane displacement, which can be determined from the variation in the magnetic flux density of the bottom magnet.

Localization has been introduced after the measurement of shear force, and note that if shear force exists, magnets displacements used for localization and normal force estimation have to be the normal force-induced displacements, which means the shear force-induced displacement in magnets 2 and 3 have to be deducted from the total displacements, then the pure-normal-force-induced displacements can be used to localize the external load (shown in Equation (S12)) and estimate the magnitude of normal force:

$$F_z = (1 + \frac{|F_p|}{\nu_f})\nu_s K_z \cdot U_z(U_{bv}, \nu_{a,X}, \nu_{a,Y}, U_p) \tag{S27}$$

where ν_f is the folding state correction coefficient to compensate the influence of the folding state caused by the existence of shear force, which have been discussed in Discuss section of main article. ν_s is the coefficient to compensate for the stiffness variation in the fabrication process, and U_z is the displacement of the top vertex in the Z direction of the cell under load, which can be calculated from the magnets displacements and attenuation coefficient.

The accuracy of the theoretical model without correction coefficients was degraded due to several factors: 1) the manufacturing process affects the mechanical properties. 2) The displacements in X and Y directions of the top vertex in the loaded cell were nonzero contradicting the assumption that the top vertex only moves in the Z direction, particularly for edge cells. 3) The SMo structure did not fold and unfold as an ideal rigid-foldable Miura-ori structure. To address these issues, several modifications were implemented: 1) A constraint frame, shown in Figure 3e in main text, was introduced to restrict the in-plane displacements of edge columns, and to mitigate external interference and potential damage to the supporting columns caused by excessive displacement. 2) Two attenuation coefficients, $\nu_{a,X}$ and $\nu_{a,Y}$, were introduced to describe the decrease in unfolding state with the increase of distance. 3) Another coefficient, the stiffness variation factor ν_s was introduced to account for variations in stiffness during the fabrication process. 4) The folding state correction coefficient ν_f was also introduced to correct the extra folding state caused by the shear forces, since the folding cannot be perfectly constrained by the top elastomer scales when shear force exists. According to Equation (S27), the estimated normal force is adjusted by a factor of $1 + |F_p|/\nu_f$, when the shear force is zero, ν_f has no effect on the estimate of normal force. As the shear force increases, $1 + |F_p|/\nu_f$ also increases, providing a more accurate estimate of the normal force. Alternatively, improving the accuracy of the fabrication, adjusting the material properties, or introducing microstructures to better constrain folding could also mitigate these issues.

Stiffness Distributions for Different Attenuation Coefficients

Different values of the attenuation coefficient will lead to different stiffness distributions. $\nu_{a,X}$ and $\nu_{a,Y}$ were assumed to be the same here. The stiffness distribution (normalized stiffness) of the theoretical model for different values of $\nu_{a,X}$ and $\nu_{a,Y}$ is shown in Figure S7. When $\nu_{a,X}$ and $\nu_{a,Y}$ equal 1, the edge cell stiffness is obviously larger than the center cell stiffness, which is a contrast to real scenarios. With the decrease of $\nu_{a,X}$ and $\nu_{a,Y}$, the differences between the stiffness of the center and edge cells decrease, then the stiffness of the center cell will be greater than the stiffness of the edge cell with the continued decrease of $\nu_{a,X}$ and $\nu_{a,Y}$, which is the real case of the stiffness distribution.

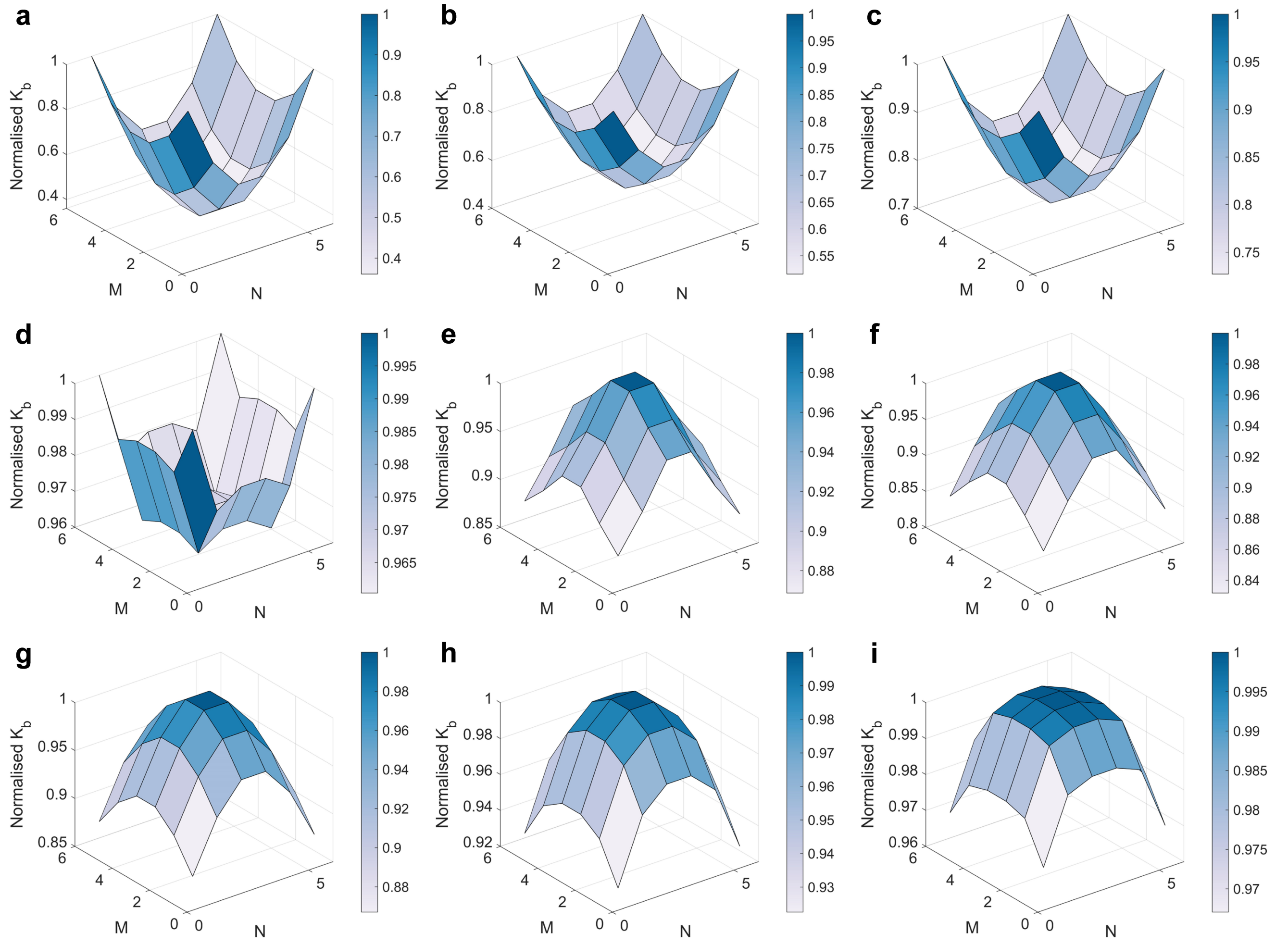

Figure S7. Theoretical model normalized stiffness distribution for different values of $v_{a,X}$ and $v_{a,Y}$. (a) $v_{a,X} = v_{a,Y} = 1$. **(b)** $v_{a,X} = v_{a,Y} = 0.9$. **(c)** $v_{a,X} = v_{a,Y} = 0.8$. **(d)** $v_{a,X} = v_{a,Y} = 0.7$. **(e)** $v_{a,X} = v_{a,Y} = 0.6$. **(f)** $v_{a,X} = v_{a,Y} = 0.5$. **(g)** $v_{a,X} = v_{a,Y} = 0.4$. **(h)** $v_{a,X} = v_{a,Y} = 0.3$. **(i)** $v_{a,X} = v_{a,Y} = 0.2$.

Calibration Process

There are 4 coefficients to be identified in the calibration process, the attenuation coefficient $v_{a,X}$ and $v_{a,Y}$, the stiffness increasing factor v_s and the folding state correction coefficient v_f.

$v_{a,X}$ and $v_{a,Y}$ will affect the stiffness distribution, and here these two coefficients are assumed to be the same according to the previous analysis. In order to know the value of $v_{a,X}$ and $v_{a,Y}$, according to the symmetry of the Miura-ori structure, the stiffness of 3 cells is measured here: cell (1,1), (2,2) and (3,3), which represents the outer layer of cells, mid layer of cells and inner layer of cells, respectively, and this is enough to get an accurate result. Then the value of $v_{a,X}$ and $v_{a,Y}$ can be chosen as the one that generates a most closely matched stiffness distribution in the theoretical model as measured values.

$v_{a,X}$ and $v_{a,Y}$ will affect not only the stiffness distribution but the absolute value of the stiffness of the theoretical model. After identifying the values of $v_{a,X}$ and $v_{a,Y}$, v_s can be acquired by making the absolute stiffness value the same as the measured value from the experiment.

The calibration of v_f is more complex than the other coefficients, as there are different in-plane stiffness for different cells because of interactions between cells, and the in-plane stiffness in different directions of the same cell are also different. Here, all the cells are also divided into three different layers: out layer, mid layer and inner layer, and cell (1,1), (2,2) and (3,3) are used to represent cells belong to these layers. The calibration of v_f is carried out in two directions: shear force in the X direction and shear force in the Y direction, and in each calibration scenario, the angle of force is set to 45 degrees, which can generate the same normal load as the shear load, and the load is applied in three different cells. Therefore, 6 coefficients have to be tested, 3 in the X direction for different layers and 3 in Y direction for different layers. Because the magnitude of the normal

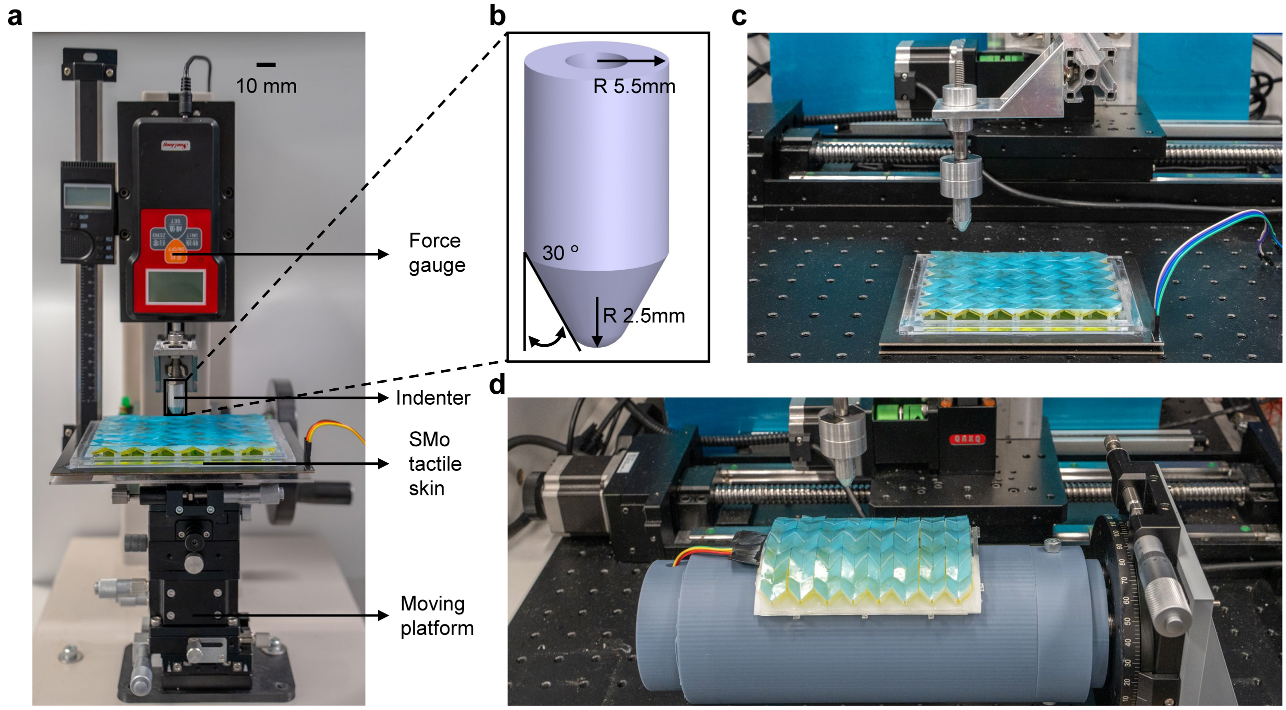

Figure S8. Sensor test and data collection platform. (**a**) Tactile skin performance test platform. (**b**) Indenter dimension data. (**c**) Machine learning data collection platform for planar case and (**d**) for curved SMo tactile skin.

force is known, the value of v_f can be identified as the one that results in the most accurate result. For the X direction, v_f is 0.6, 0.75 and 0.9 for the outer layer, the mid layer, and the inner layer, respectively, and the counterparts in the Y direction are 0.9, 1 and 1.1. The higher value of v_f indicates a greater influence of the shear load on the normal load estimation, which means that the stiffness of the in-plane in the X direction is smaller than in the Y direction.

Tactile Skin Characterization and Data Collection

The developed tactile skin was evaluated using a commercial force gauge (SanLiang Corp.) and a moving platform that allows movement in three directions, rotation about one axis and tilt in two axes, as depicted in Figure S8. The force-displacement curve was obtained using a compression testing machine (Instron 68SC-05). Furthermore, the machine learning data collection platforms for the planar and curved cases are illustrated in Figures S8b and c.

Recovery Time

The recovery times for cells (1,1), (2,2), (3,3), (4,4), (5,5) and (6,6) are shown in Figure S9 (cell (1,1) is shown here again for convenience).

Magnet Material Characterization and Simulation

The magnetization loop is shown in Figure S10, which indicates that the remanent magnetization M_r is 0.804 emu$/$mm^3 at $H = 0$, and the intrinsic coercivity H_{ci} is -5227.78 Oe at $M = 0$. Consequently, the magnetization m_0 introduced earlier can be calculated as $m_0 = M_r \times 10^6$ A/m, according to the definition of magnetization (magnetic moment per unit volume).

To illustrate the effect of the SMo structure, we performed a mechanical-magnetic coupling simulation of a soft centripetal magnet with dimensions identical to those of the SMo tactile skin. The simulation setup is depicted in Figure S10a. A displacement boundary condition of -2 mm was applied at the center of the upper surface and the magnetic flux density was measured at two distinct points: (0,0,-3), located directly below the deformation area, and (100,100,-3), positioned away from the deformed region. Magnetic flux density changes are shown in Figure S10b, and the magnetic flux density distributions before and after deformation are illustrated in Figure S10c and d.

The results reveal a notable change in the magnetic field near the deformed area. Conversely, at a location distant from the deformation point, such as (100,100,-3), there is minimal observable change in magnetic flux density, which is not detectable

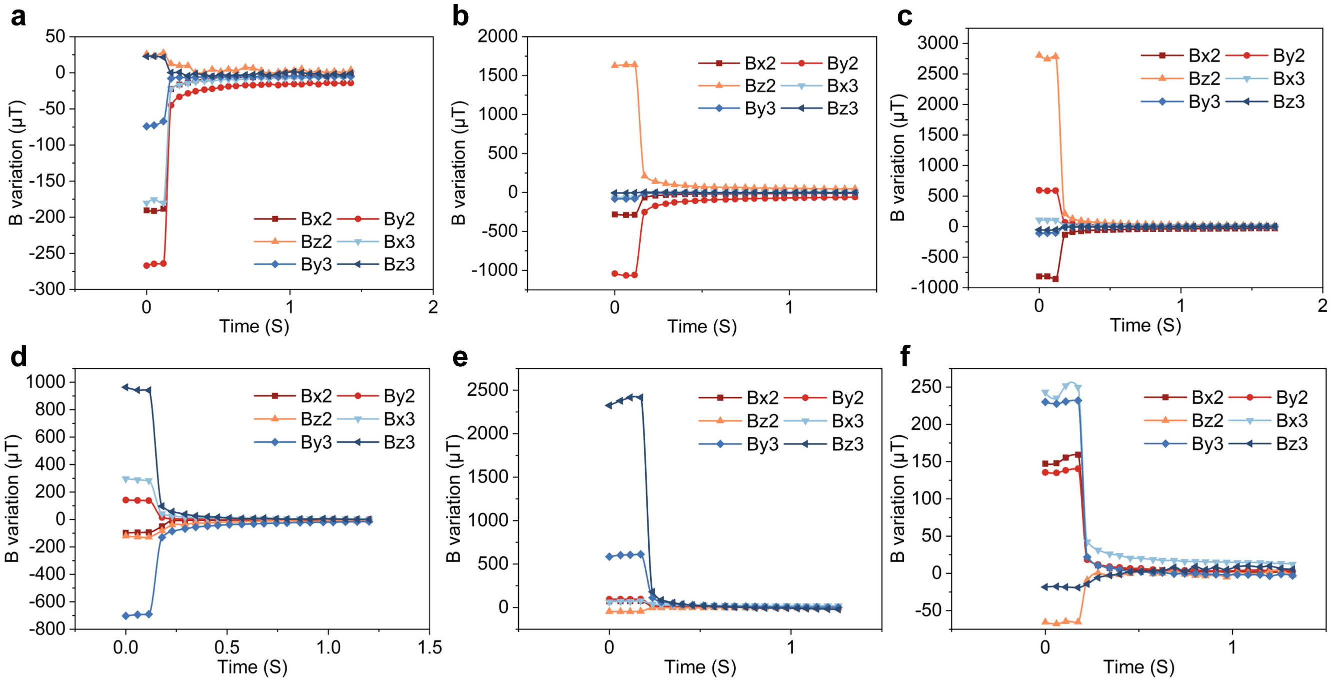

Figure S9. Recovery time when a load of 1N applied on different cells. (a) On cell (1,1), for convenience, which is shown again here. **(b)** On cell (2,2). **(c)** On cell (3,3). **(d)** On cell (4,4). **(e)** On cell (5,5). **(f)** On cell (6,6)

with the current Hall sensor (MLX 90390). The magnetic field measurement point was chosen so that the distance between it and the upper surface of the magnet in the z direction matches the thickness of our SMo tactile skin. Increasing this distance could enhance the visibility of magnetic variations, but would also considerably increase the thickness of the tactile system and potentially introduce other issues.

Signal Matrix Values for Different Zones

The values of the signal matrix for different zones are shown in Table S1.

Zone	dS2x	dS2y	dS3x	dS3y
Z.1	-	-	-	-
Z.2	-	-	+	-
Z.3	+	-	+	-
Z.4	-	+	-	-
Z.5	-	-	+	-
Z.6	+	+	+	-
Z.7	-	+	-	+
Z.8	-	+	+	+
Z.9	+	+	+	+

Table S1. Values of signal matrices for different zones.

Machine Learning Performance with Preprocessed Input

After training the machine learning model, we have also tested its performance when both normal and shear forces are applied. The machine learning algorithm was developed for pure normal force estimation, and the existence of the shear load will affect the performance. Therefore, the input was preprocessed before sending to the machine learning algorithm. The preprocess was:

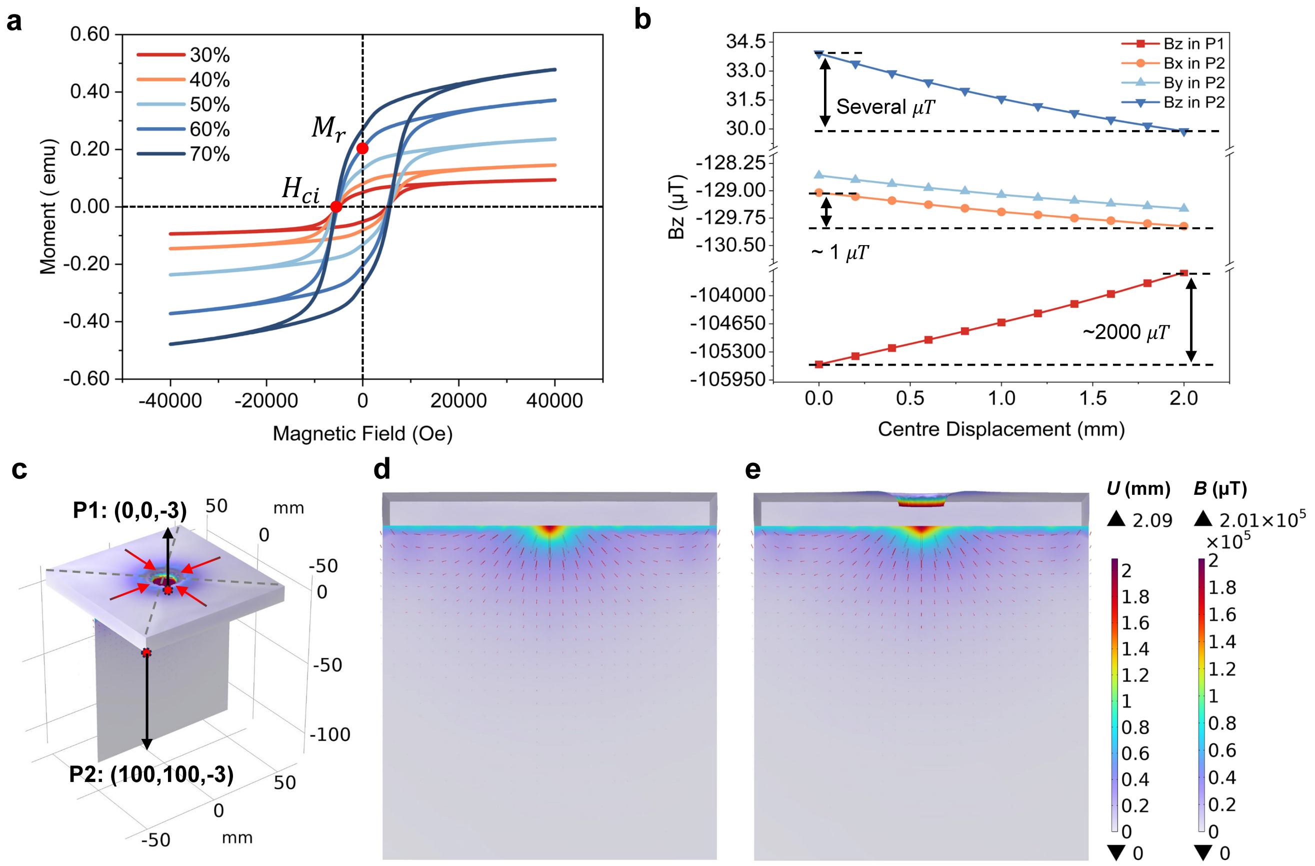

Figure S10. Magnet material characterization and soft magnet simulation. (**a**) Magnetic hysteresis curve. (**b**) Magnetic flux density change of whole soft magnet for two different points. (**c**) Simulation set up. (**d**) and (**e**) Deformation field (U) and magnetic flux density field (B) of whole soft magnet before and after deformation, respectively.

at first, shear forces were measured from the signal readout of the lower surface hall sensor, and then the shear force-induced displacement in the upper surface hall sensors can be calculated, thus the shear force-induced magnetic flux density variation. Then it will be deduced from the signals from two upper Hall sensors; therefore, the pure normal force induced magnetic flux density variation can be calculated and used as the input of machine learning. In addition, the correction term $(1+\frac{|F_p|}{v_f})$ used in the theoretical model, was also applied here (multiplied with the estimated normal force). Because signals from the lower hall sensor were used for the measurement of the shear force, the 6-input model was used for localization and estimation of the normal force.

When without this preprocess, the normal force estimation was quite inaccurate. When shear force-induced influence was deduced, normal force estimation becomes more accurate, as shown in Figures S11a and d. However, the localization accuracy was not satisfied even the localization error decreased in some scenarios, as the results without and with preprocess are shown in Figure S11b, c, e and f. It is obvious that for most of the cells tested, the localization error was reduced with the use of preprocessed input, but they were still large. One of the possible reasons for this phenomenon is that the corrections from the theoretical model are incompatible with the machine learning algorithm. Therefore, in order to solve this issue, the algorithm needs to be further investigated to find a more suitable correction or calibration method.

Discussion about Multi-Point Touch

Theoretically, for the sensor of the present configuration, there are a total of nine known quantities, and if, in the case of measuring only normal force, there are three unknown quantities at each point, the coordinates X, Y, and the force magnitude F. In the case of two point normal force touch with current planar configuration, there are 6 unknowns, and totally 9 signals can be collected; therefore mathematically it can be solved.

If using a machine learning algorithm to locate and estimate the force, data collection will be quite complicated. The data from the sensor are to be collected when two loads are applied at the same time, and there are many different combinations of

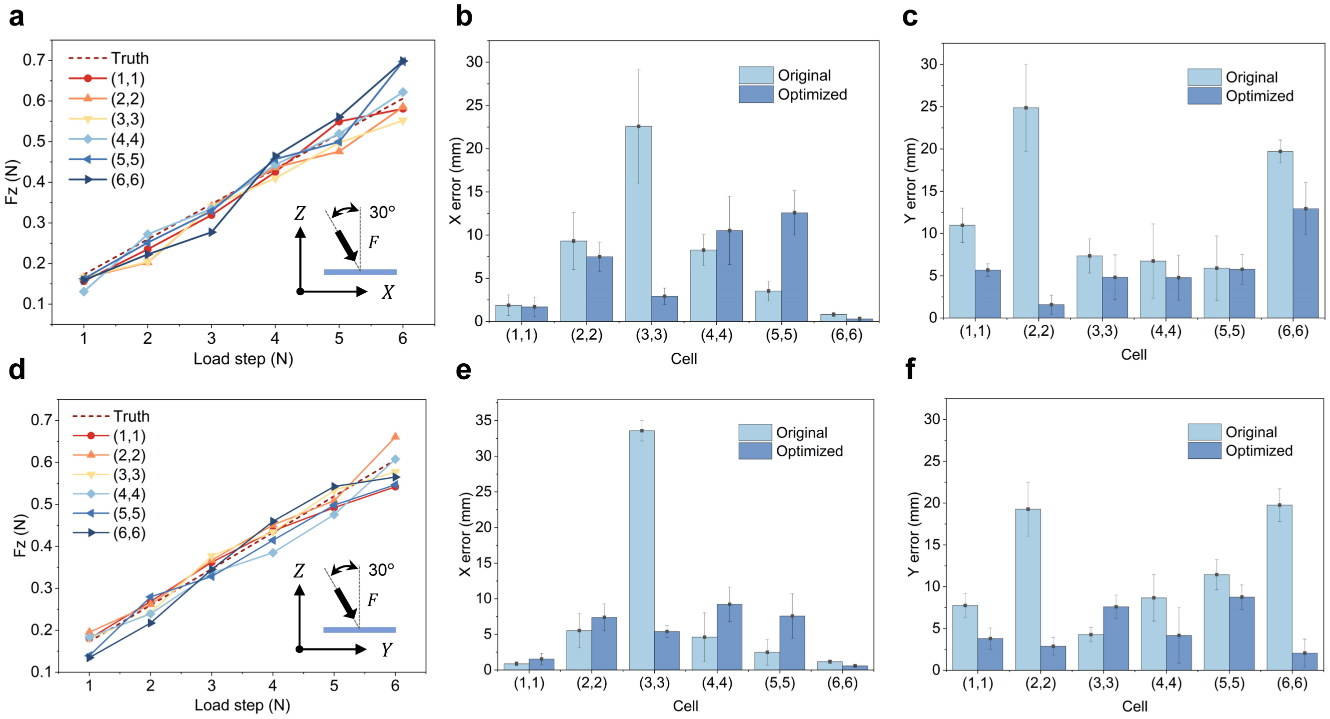

Figure S11. Machine learning performance with preprocessed input when both normal force and shear force are applied. **(a)** Normal force estimation performance using preprocessed input when shear force is applied on X direction. **(b)** and **(c)** X and Y localization error before and after optimization when shear force is applied on X direction. **(d)** Normal force estimation performance using preprocessed input when shear force is applied on Y direction. **(e)** and **(f)** X and Y localization error before and after optimization when shear force is applied on Y direction.

loads applied at the same time at two points. For the current configuration, single-point load would require 825 (25 by 33) data for each magnitude of force if the current spacing of the data collection is followed, but for two points touch, 680625 (825 by 825) data is required if the spacing is kept the same and the two forces are of the same magnitude. If different combinations of force between two points are to be considered, and assuming we are going to apply the force from 0.1N to 1N with an incremental of 0.1N, thus the data set should be 68062500 ($680625 \times 10 \times 10$), which is quite a large amount of data.

In addition, for the current machine neural network, the dimensions of input and output are prescribed, which means that for single point touch, the output is a matrix of 1×3 and for two point touch, the output is a 1×6 matrix. In the sensing process, the current algorithm cannot switch automatically, which means that it cannot tell if it is a single point or multiple points under load. Therefore, if we want to achieve multipoint touch, algorithm improvement is required. Additionally, for most human-robot interaction scenarios, one point touch is enough.

Curved Surface Tessellation

The in-plane stretching energy for the Miura-ori structure can be demonstrated as[3]:

$$W_{stretching} = C_1(I_1 - 3) \tag{S28}$$

where C_1 is a material constant equal to half the initial shear modulus, and I_1 is the first invariant (trace) of the right Cauchy-Green deformation tensor, given by $I_1 = \lambda_1^2 + \lambda_2^2 + \lambda_3^2 = \mathrm{tr}(\mathbf{C}) = C_{II}$. The out-of-plane bending stiffness can be demonstrated by[4]:

$$W_{bending} = B(k_1^2 + k_2^2) = B \cdot b_{\alpha\beta} b_{\alpha\beta} \tag{S29}$$

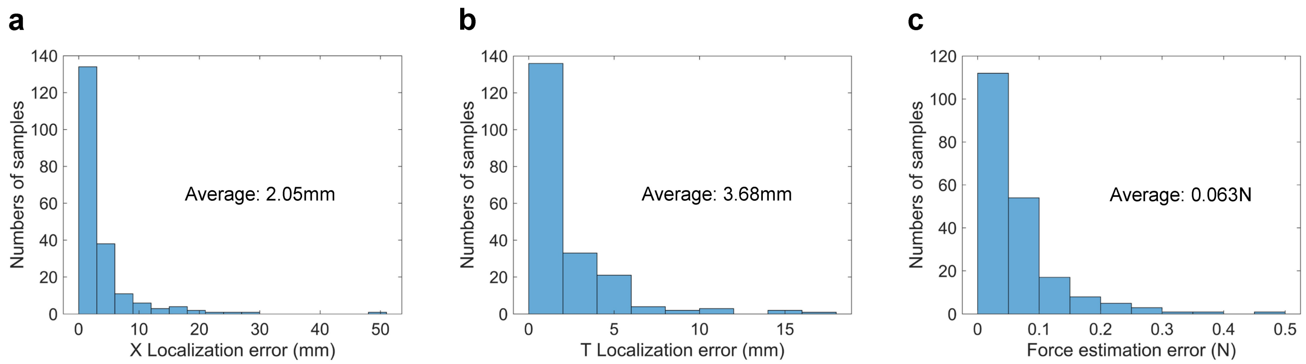

Figure S12. Performance of curved SMo tactile skin. **(a)** X localization error distribution of 200 tests. **(b)** T localization error distribution of 200 tests. **(c)** Force estimation error distribution of 200 tests.

where k_1 and k_2 are two principal curvatures. Therefore, the total elastic energy of the Miura-ori structure is[5]:

$$W[\mathbf{x}(\mathbf{X})] = \int_A \left[\tau(C_{II} + \frac{1}{\det(\mathbf{C})} - 3) + b_{\alpha\beta}b_{\alpha\beta}\right] dA \tag{S30}$$

where τ is a coefficient used to adjust the relative importance of in-plane stretch and out-of-plane bending. In fact, the tessellation design problem is a minimization problem of the total elastic energy for different processes. One process is the attachment process $\mathbf{x}(\mathbf{X})$, and the other is the in-plane folding process $\mathbf{X}(\mathbf{X_0})$. Thus, the tessellation design problem ultimately becomes:

$$\min_{\mathbf{X}(\mathbf{X_0})} \left(\min_{\mathbf{x}(\mathbf{X})} W[\mathbf{x}(\mathbf{X}(\mathbf{X}_0))]\right) \tag{S31}$$

In addition, the size of the panel and other geometry parameters can also be adjusted by introducing different coefficients[5].

Demo of Human-Machine Interaction for Rapidly Changing Contact Pressures

In order to quantitatively characterize the performance of the curved SMo tactile skin, we performed 200 localization and external force estimation tests, the results are shown in Figure S12. From the results, the average localization error in X and T direction are 2.05mm and 3.68mm, respectively, showing the accurate localization capability; the average force estimation error is 0.063N, also showng its excellent force estimation ability. If further improvements in accuracy are desired, they can be achieved by increasing the training set or improving the algorithm.

In addition, to illustrate the response of tactile skin to rapid change in external loads, we have developed a visualization interface using MATLAB, and performed a demo of interaction with the robotic arm. In this demo, a threshold of 0.5N was set, if external load is less than 0.5N, the robotic arm will not move; if it is larger than 0.5N, the robotic arm will translate along the direction of external load. Demo results are shown in Figure S13 and Movie S2.

In the demo video, when the external force is less than 0.5N, the tactile skin is able to continuously and rapidly localize the external force and calculate its magnitude, and when the external force is larger than 0.5N, the control signal will be sent to the robotic arm, and then the robotic arm will move along the direction of the external force, showing the sensor's ability to respond sensitively to rapidly changing external loads.

Stability testing of sensors in different environments

To explore the stability of SMo tactile skin in different environments, we have tested its performance in different environments, including different temperatures and different humidity.

Temperature affects the performance of the SMo tactile skin mainly in two aspects, firstly, the structure, mainly the bonding between the panels and the middle film will be affected. Secondly, magnets' performance will be affected by different. Therefore, we tested the the structure stability and magnet's magnetic flux density variation under different temperatures.

The performance of layered Miura-ori after being placed at different temperatures (50°C and 75°C) for 24 hours is shown in Figure S14. From Figure S14c, after placed in 50°C for 24 hours, there is almost no degradation of folding and unfolding

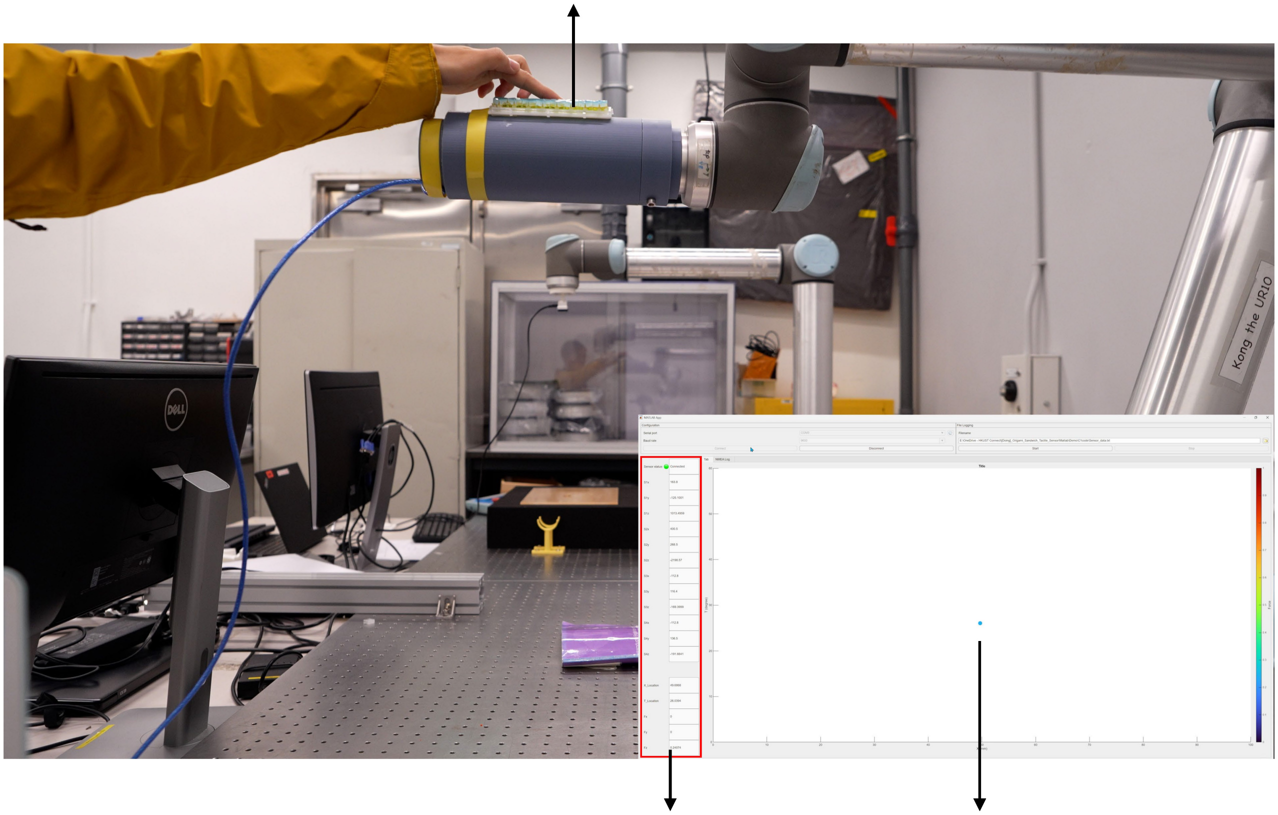

Figure S13. Human-machine interaction demo with prescribed external force threshold.

performance (no debonding). However, after placing in 75 ° C for 24 hours, when folding, obvious debonding was observed between the panels and the middle film, as shown in Figure S14e. To solve the issue of debonding of layered Miura-ori at high temperature, we also explore the performance of hot press fabrication. The schematic of hot press are shown in Figure S14b, the layered Miura-ori are placed between two hot plate, then these plate are heated to 320°C, and two hot plates will squeeze the sample, making a tight bond between the different layers. The performance of hot-pressed SMo is shown in Figure S14f, g and h. It is obvious with the help of hot press, even placed at 75°C for 24 hours, there is no debonding between different layers.

Therefore, according to different application scenarios and usage environments, different fabrication processes can be selected to make the structure easy to manufacture while maintaining a high degree of stability.

The magnetic flux density value of magnet under different temperature is shown in Figure S15. In this test, magnet was fixed on a sensor platform, and the platform in placed into the oven with prescribed temperature, as shown in Figure S15a. In Figure S15b, when the temperature increases, the magnetic flux density decreases in all three directions, and the variations were almost linear (the amount of variation is approximately 5% per 10 degrees Celsius). (Note that the MLX90393 is capable of measuring magnetic fields as well as temperature, and it has built-in temperature compensation. In this test, the reference temperature for temperature compensation is set to 25 degrees Celsius). Therefore, for different temperature environments, the external temperature can be known with the help of the MLX90393, and then with the help of the magnet's temperature attenuation characteristics, the sensor signal can be corrected accordingly to ensure the performance.

For the performance of the origami structure in different humidity levels, we soaked the origami structure in water for 24 hours, then took it out to fold and unfold it, and there was no noticeable debonding appearing. The humidity had no effect on the magnetic field distribution of the magnets, nor did the light. In addition, for both humidity and waterproofing related issues, they can be solved by coating the device surface with a parylene film[6].

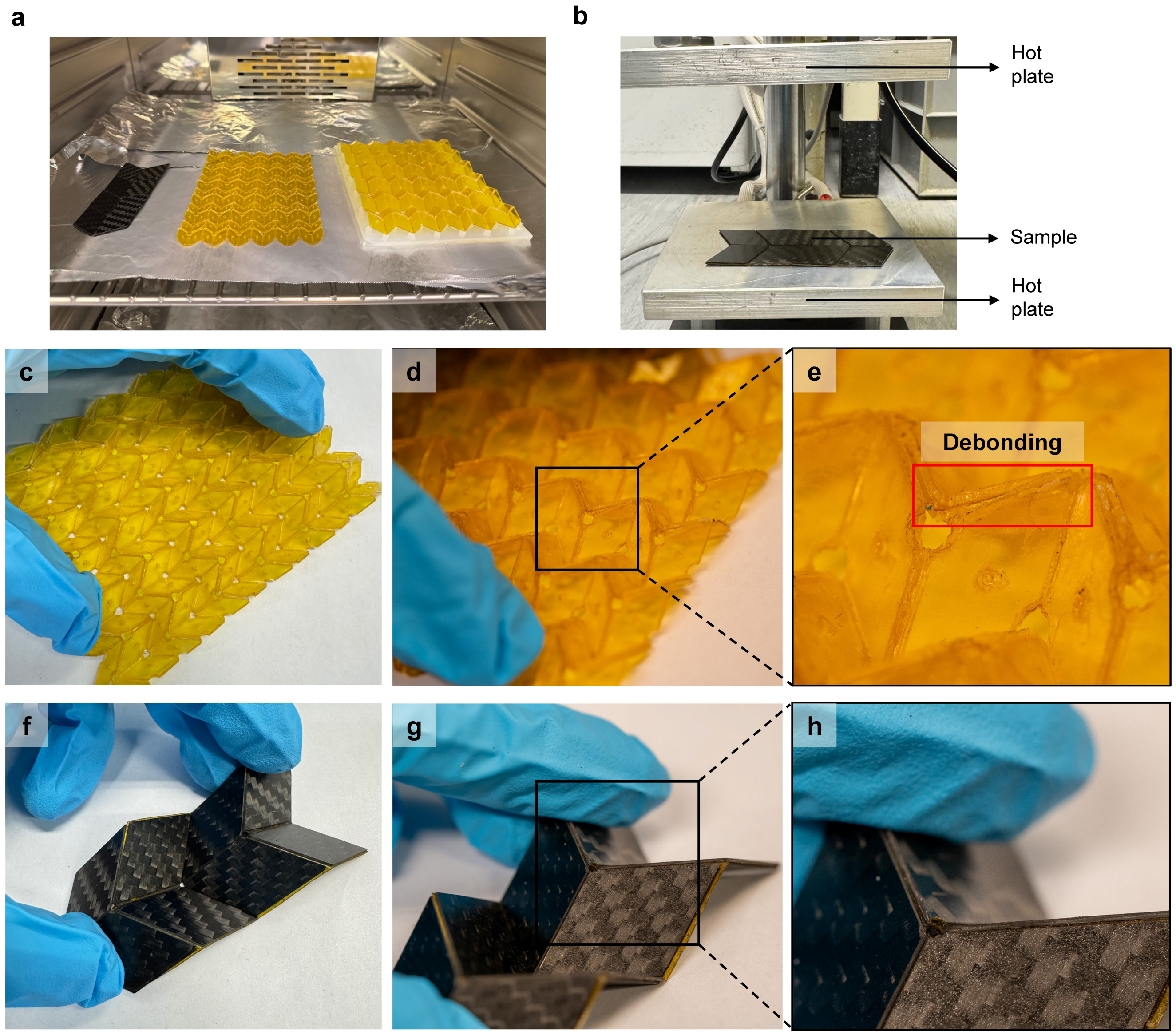

Figure S14. Testing of structural stability at different temperatures. **(a)** Experiment setup, samples were placed in an oven which can be set to different temperatures. **(b)** Schematic of hot press fabrication. **(c)** Folding performance of layered Miura-ori after placed in the oven of 50°C for 24 hours. **(d)** Folding performance of layered Miura-ori after placed in the oven of 50°C for 24 hours. **(e)** Zoom in of (d). **(f)** Folding performance of layered Miura-ori with hot press fabrication after placed in the oven of 75°C for 24 hours. **(g)** Folding performance of layered Miura-ori with hot press fabrication after placed in the oven of 75°C for 24 hours. **(h)** Zoom in of (g).

A Possible Structure to Solve The Issue of Limited Sensing Points

In order to solve the issue of limited sensing point, a possible solution is to cover all cells using flat panels, as shown in Figure S16. In this way, the force acting anywhere above each cell will become the displacement of the top vertex in the vertical direction. Once external load is applied, the bar connected with the top cover panel will move downward, and thus its in-plane displacement will be constrained, which will make the cell under load more conformance with the assumptions (the top vertex of the cell under load only moves in the Z direction). For other cells, their translation will not be affected because the vertical bar connected to the top cover panel is not initially constrained by the translation constraint. Note that this structure will not increase the resolution, and it only increases the sensing point from the top vertices to anywhere above the cell, so machine learning is not compulsory for this structure and the theoretical model is enough.

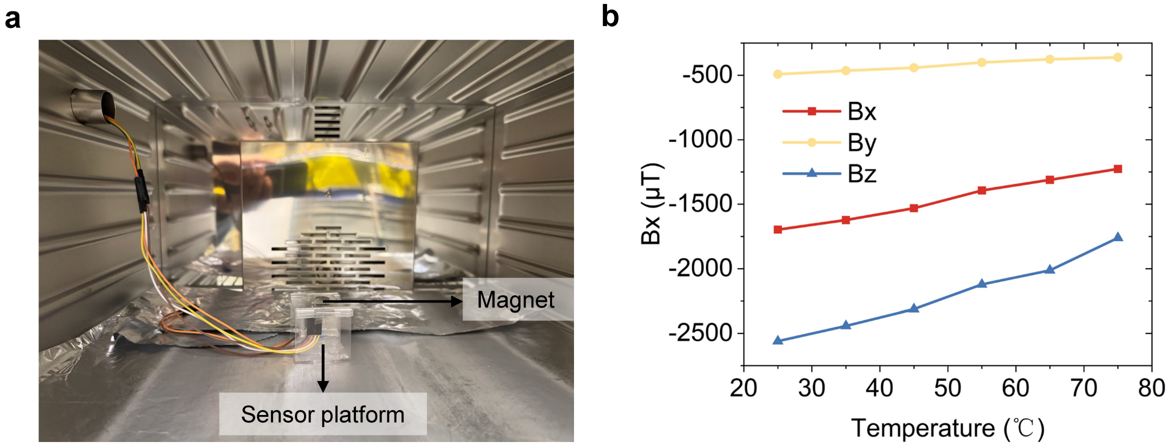

Figure S15. Influence of temperature on the magnet. **(a)** Experiment setup. **(b)** Magnetic flux density of magnet in different temperature.

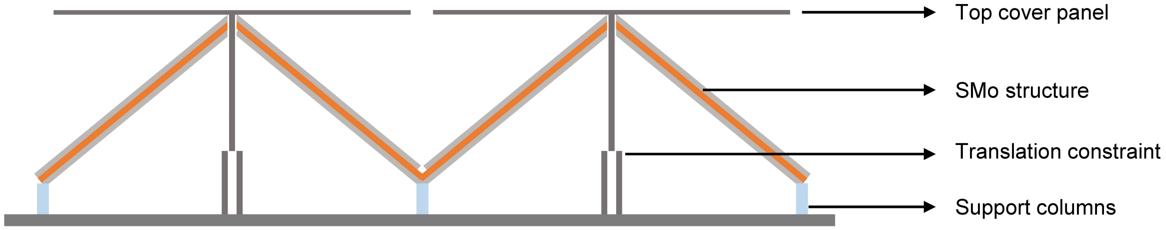

Figure S16. A possible structure to solve the limited sensing point issue without machine learning.

References

S1. Dai, H. *et al.* Split-type magnetic soft tactile sensor with three-dimensional force decoupling. *Adv. Mater.* 2310145 (2023).

S2. Wei, Z. Y., Guo, Z. V., Dudte, L., Liang, H. Y. & Mahadevan, L. Geometric mechanics of periodic pleated origami. *Phys. review letters* **110**, 215501 (2013).

S3. Holzapfel, G. A. Nonlinear solid mechanics: a continuum approach for engineering science (2002).

S4. Guggenheimer, H. W. *Differential geometry* (Courier Corporation, 2012).

S5. Deng, Y. *et al.* Curved display based on programming origami tessellations. *Microsystems & Nanoeng.* **7**, 101 (2021).

S6. Sui, W., Duvieusart, M. S., Zhao, J., Tai, Y.-C. & Lee, Y.-K. Comparative study of the viscoelasticity of parylene thin films for mems using nano-dma and molecular dynamics. In *2017 IEEE 30th International Conference on Micro Electro Mechanical Systems (MEMS)*, 468–471 (IEEE, 2017).
